# High fidelity DNA strand-separation is the major specificity determinant in DNA methyltransferase CcrM’s catalytic mechanism

**DOI:** 10.1093/nar/gkad443

**Published:** 2023-06-16

**Authors:** Olivia Konttinen, Jason Carmody, Martin Kurnik, Kenneth A Johnson, Norbert Reich

**Affiliations:** Biomolecular Science and Engineering, University of California, Santa Barbara, Santa Barbara, CA, USA; Chemistry and Biochemistry, University of California, Santa Barbara, Santa Barbara, CA, USA; Chemistry and Biochemistry, University of California, Santa Barbara, Santa Barbara, CA, USA; Life Sciences Interdisciplinary Graduate Program, Department of Molecular Biosciences, University of Texas, Austin, TX, USA; Biomolecular Science and Engineering, University of California, Santa Barbara, Santa Barbara, CA, USA; Chemistry and Biochemistry, University of California, Santa Barbara, Santa Barbara, CA, USA

## Abstract

Strand-separation is emerging as a novel DNA recognition mechanism but the underlying mechanisms and quantitative contribution of strand-separation to fidelity remain obscure. The bacterial DNA adenine methyltransferase, CcrM, recognizes 5′GANTC′3 sequences through a DNA strand-separation mechanism with unusually high selectivity. To explore this novel recognition mechanism, we incorporated Pyrrolo-dC into cognate and noncognate DNA to monitor the kinetics of strand-separation and used tryptophan fluorescence to follow protein conformational changes. Both signals are biphasic and global fitting showed that the faster phase of DNA strand-separation was coincident with the protein conformational transition. Non-cognate sequences did not display strand-separation and methylation was reduced > 300-fold, providing evidence that strand-separation is a major determinant of selectivity. Analysis of an R350A mutant showed that the enzyme conformational step can occur without strand-separation, so the two events are uncoupled. A stabilizing role for the methyl-donor (SAM) is proposed; the cofactor interacts with a critical loop which is inserted between the DNA strands, thereby stabilizing the strand-separated conformation. The results presented here are broadly applicable to the study of other N^6^-adenine methyltransferases that contain the structural features implicated in strand-separation, which are found widely dispersed across many bacterial phyla, including human and animal pathogens, and some Eukaryotes.

## Graphical Abstract

**Graphical Abstract:**
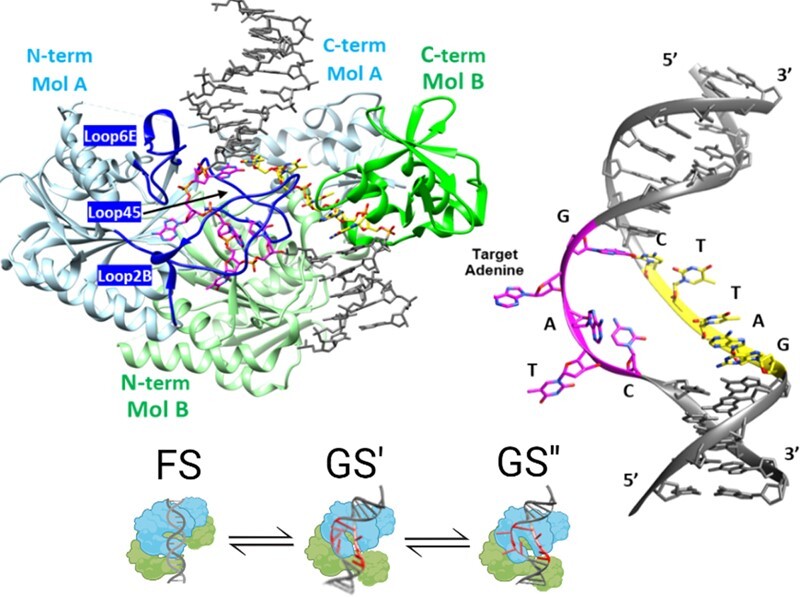

## INTRODUCTION

The protein-mediated separation of DNA strands, resulting in specific protein-DNA interactions largely with only one of the two strands, is emerging as a novel DNA recognition mechanism (1,2). Despite numerous cocrystal structures, the events leading up to the strand-separated structures remain enigmatic (3,4). Cell-cycle regulated DNA Methyltransferase (CcrM), a β-class DNA methyltransferase (MTase) (5) from *Caulobacter crescentus* is responsible for the maintenance methylation of 4515 5′GANTC3′ sites in the bacterial chromosome during cell-cycle division (6). CcrM DNA methylation is an example of bacterial epigenetic regulation, where the *N*^6^-methyladenine epigenetic mark is essential for the efficient transcription of numerous genes involved in cell-cycle progression in *Caulobacter crescentus* (7,8).

CcrM methylates *N*^6^-adenine within 5′GANTC3′ recognition sites using the cofactor S-adenosyl-L-methionine (SAM) (9). CcrM does not have a cognate restriction endonuclease, therefore it is considered an orphan MTase (10). CcrM and other orphan MTases that recognize 5′GANTC′3 sites have an unusual 83-amino acid residue C-terminal domain that interacts with the phosphate backbone of the nontarget strand (11). CcrM homologs are widely dispersed amongst human and/or animal pathogens including *Brucella melitensis* (12), *Rhodobacter massiliensis* (13), *Pannonibacter phragmitetus* (14), *Inquilinus limosus* (15), *Haematospirillum jordaniae* (16), *Trichomonas vaginalis* (17), *Moraxella lincolnii* (18), *Campylobacter sputorum* (19), *Mycoplasma falconis* (20) and *Helicobacter pylori* (11,21). Substitution of conserved residues in the C-terminus of CcrM negatively impacts both DNA recognition and strand separation (11).

**Figure 1.**
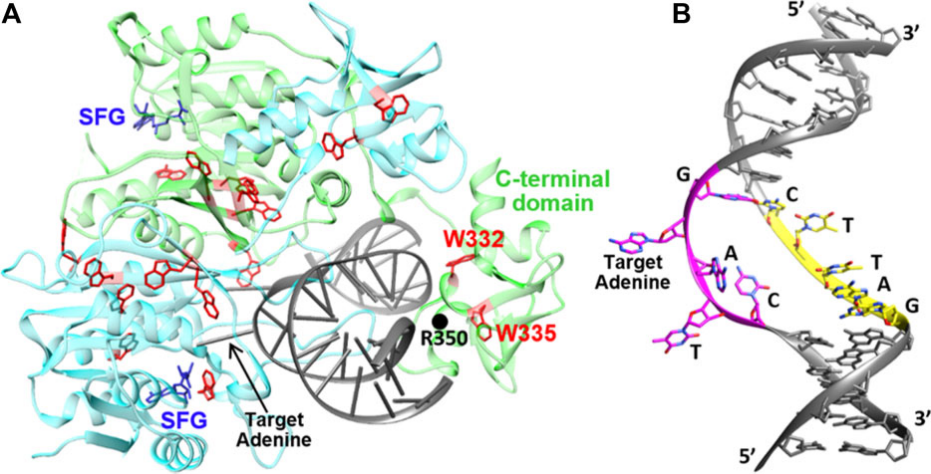
The CcrM cocrystal structure depicts GS^II^ in our kinetic model. (**A**) The structure shows the CcrM dimer with 18 Trp residues (red), strand-separated DNA (grey), and two Sinefungin molecules (SFG; blue). R350, W332 and W335 are located in the C-terminal 83-residue segment of monomer B (green), and the N-terminus of monomer A (cyan) is positioned to catalyze methyl transfer on the target adenine. (**B**) DNA strand separation at the recognition site. The target strand recognition site is colored magenta and the nontarget strand recognition site is colored yellow. Four of the five basepairs of the recognition site are disrupted. The 5’ G of the target strand maintains base pairing to the 3’ C of the nontarget strand. Base-stacking interactions between non-target strand bases are maintained, while base-stacking of target strand bases is lost. Molecular graphics were made with UCSF Chimera.

CcrM is unusually discriminating, compared to other DNA MTases, having at least 10^6^-fold loss in specificity for non-cognate recognition sequences (e.g. 5′AANTC3′ compared to 5′GANTC′3) (22). In contrast, another orphan DNA MTase, Dam does not strand-separate and has orders of magnitude less discrimination, as do Type II R/M DNA MTases (22). The CcrM-DNA-sinefungin cocrystal structure (Figure 1) reveals that four of the five basepairs within the 5′GANTC′3 recognition site are completely disrupted from B-form helical DNA (23), and the DNA strand targeted for methylation is no longer base-paired within the 5′GANTC′3 recognition site. The non-target strand is less perturbed, retaining base-stacking (Figure 1B).

We previously used Pyrrolo-dC (PydC), a fluorescent analog of deoxycytidine, to track the strand-separation event using equilibrium fluorescence measurements (11). DNA harboring PydC maintains B-form helical structure and basepairs to dG as a normal deoxycytosine nucleotide (24–29). In contrast to dC, however, PydC is highly fluorescent, with excitation and emission wavelengths of 350nm and 450nm, respectively. PydC fluorescence intensity is quenched due to base stacking and base-pairing interactions so it provides an optimal signal to monitor the transition from dsDNA to ssDNA (24–29). Upon CcrM binding, an increase in PydC fluorescence is observed, monitoring the transition from dsDNA to ssDNA (11). Similar equilibrium PydC results were obtained with the CcrM ortholog from *Brucella abortus*, as well as the β-class DNA MTase HinfI, both of which have the 83-residue C-terminal domain and methylate both ssDNA and dsDNA (11). Thus, the PydC assay shows similar protein-dependent fluorescence changes with enzymes that are functionally and structurally similar to CcrM (11). In contrast, no fluorescence changes are observed with M.HhaII, which recognizes the same site (5′GANTC′3), lacks the C-terminal domain, and which shows > 300 times less activity with ssDNA (11); CcrM methylates ssDNA as efficiently as dsDNA (30).

Expanding on our previous equilibrium measurements, here we use stopped-flow fluorescence to monitor the kinetics of a protein conformational transition using intrinsic tryptophan fluorescence and the DNA strand separation event using PydC fluorescence. We measured these transitions for WT CcrM with cognate and noncognate DNA and for CcrM variant (R350A) with cognate DNA. Our results provide a basis for investigating the ordered events that lead up to the strand-separated CcrM-DNA complex and shed light on which events contribute to the enzyme's DNA sequence-specificity. For example, conformational transitions such as base-flipping can be rate-limiting and contribute significantly to sequence discrimination (31), so the first questions to address is whether DNA strand separation by CcrM is rate-limiting and whether it is readily reversible.

In spite of numerous structural studies demonstrating changes in enzyme structure after substrate binding, the fidelity contribution of substrate-induced conformational changes has been controversial. For example, Fersht argued that a two-step binding contributes the same free energy change as a one-step binding mechanism and therefore the two-step induced-fit pathway cannot add anything to selectivity beyond a simple binding step (32). Moreover, Warshel argued that a conformational change cannot influence specificity unless it is rate-limiting (33). Despite these theoretical arguments, comprehensive kinetic analysis of the conformational changes preceding nucleotide incorporation by moderate to high fidelity DNA polymerases finally showed that the nucleotide-induced change in structure is the primary determinant of fidelity even though it is not rate-limiting (34–37). Accordingly, in this study we provide direct measurements of the rate constants governing strand-separation and subsequent methylation in order to define the role of DNA strand separation in fidelity of CcrM. We show that enzyme-DNA interactions leading to strand separation are the primary determinant of fidelity even though the rates of strand separation are much faster than DNA methylation.

## MATERIALS AND METHODS

### Dna

DNA substrates were obtained from Integrated DNA Technologies and the Yale Keck Oligo Synthesis Facilities. The oligos were annealed at 95°C for 5 min in 10 mM Tris–HCl, 50 mM NaCl, 1 mM EDTA, pH 8 and subsequently cooled passively to room temperature. Annealing was analyzed by 10% native PAGE. Gels were imaged on a Bio-Rad Gel Doc EZ Imager. Densitometry was performed with FIJI ImageJ which determined a >98% annealing success.

### Site-directed mutagenesis

Variant plasmids were constructed using the Agilent Quickchange Lightning Site-Directed Kit. The primers used in the PCR reactions are described in previous work (11). Variant plasmids were transformed into XL10 Ultracompetent *Escherichia coli* cells (Agilent) and the plasmid isolated using an Agilent Mini Prep Kit. Plasmids were sequenced by the Berkeley DNA Sequencing Facility; confirmed plasmids were transformed into the New England Biolabs Nico21 (DE3) *E. coli* cells for protein production.

### Protein purification

Wild type CcrM and variant plasmids containing kanamycin resistance were cloned as described previously (30). Protein production and purification was also previously reported (11), with slight modifications. Specifically, plasmids were transformed into NiCo21(DE3) cells (New England Biolabs) and grown overnight at 37°C on LB-agar plates with 30 μg/ml kanamycin. A single colony was collected and grown for 16 h in Luria Broth (LB) medium at 37°C on a New Brunswick G10 Gyrotory shaker at 230 rpm. One-liter cultures containing 30 μg/ml kanamycin were then inoculated with the 16 hour pre-culture and grown at 37°C at 230 rpm until an OD_600_ of 0.9–1.0. The cultures were placed on ice for 10 min to facilitate their reaching of room temperature, followed by CcrM expression induced with 1.68 mM isopropylthio-β-galactoside (IPTG) at 225 rpm and 25°C for 3 h. Cells were pelleted at 4°C, 5000 rpm for 15 min in a J2-21 centrifuge (Beckman Coulter) with a JA-10 rotor and stored at –80°C. Cells were resuspended in 50 ml of lysis buffer (50 mM HEPES, 400 mM NaCl, 10% glycerol, 10 mM imidazole, pH 8.0). After the addition of phenylmethylsulfonyl fluoride (PMSF) to a concentration of 1mM, the solution, maintained at below 4°C in a water-ice slurry, was sonicated with a Branson digital sonifier at 70% amplitude for 1.5 min in 2 second increments. The lysate was cleared by centrifugation at 4°C with a JA-20 rotor at 11000 rpm for 1 hour, and the supernatant was purified at 1 ml/min on a GE 1 ml HisTrap column using an ÄKTA Start FPLC system. The bound protein was washed with 9.5 column volumes of lysis buffer and eluted with a linear gradient from 10 mM to 250 mM imidazole over 9 column volumes collected in 30 1 ml fractions. Pure CcrM fractions were identified by SDS-PAGE, and pooled, concentrated, and dialyzed over four buffer exchanges into 50 mM HEPES, 400 mM NaCl, 1mM dithiothreitol (DTT), 0.5 mM ethylenediaminetetraacetic acid (EDTA), 10% glycerol, pH 8.0 using 10 kDa cutoff Amicon Ultra 15ml centrifugal filters. The pure protein was then stored at -80°C in 25 mM HEPES, 200 mM NaCl, 0.5 mM DTT, 0.25 mM EDTA, 25% glycerol, pH 8.0. Enzyme concentrations reported throughout this work refer to the monomer concentration.

### Equilibrium tryptophan fluorescence

Equilibrium tryptophan (Trp) fluorescence measurements were performed at room temperature on a Horiba Fluoromax-4 spectrofluorometer using 296 nm excitation wavelength and 1 nm slit size. Emission data was collected at 311 nm to 430 nm using 5 nm slit size. Samples were kept on ice and allowed to equilibrate to room temperature for 5–10 min before measurements were taken. All readings were performed in 100 mM HEPES, 1 mM EDTA, 20 mM NaCl, 2 mM DTT, pH 8. Measurements were then taken in quintuplet for the buffer, additions of protein (500 nM, monomer concentration), addition of sinefungin (SFG) (60 μM), and addition of cognate or non-cognate DNA (5 μM). Each measurement and background were averaged, and statistical outliers were eliminated. Statistical outliers were defined based on the maximum signal for each scan that was outside of the lower or upper bounds. The lower bound = Q1-1.5*IQR and the upper bound = Q3 + 1.5*IQR, where IQR = inner quartile range (38). Five scans were taken, and discarded reads were not replaced. The cognate sequence is 5′-TCACTGTACTCTGACTCGCCTGACATGAC-3′ and the non-cognate sequence is 5′-TCACTGTACTCTAACTCGCCTGACAGAC-3′. Underlined bases indicate the recognition sequence of CcrM.

### Kinetic measurements by trp fluorescence

Kinetic constants were measured on an Applied Photophysics SX.18MV stopped-flow spectrometer (Leatherhead, UK) temperature controlled to 22 ± 1 °C, using 296 nm excitation and a 320 nm emission cutoff filter. Final concentrations after 1:1 mixing were 500 nM enzyme, 60.0 μM SAM (*S*-adenosyl-l-methionine), and DNA varied 2.5, 5.0, 7.5 and 10 μM, and the buffer composition was the same as in the equilibrium fluorescence experiments. Kinetic traces were collected in triplicate and averaged. The first 3ms were truncated out of each trace to account for the deadtime of the stopped-flow instrument (Supplementary Figure 1). Data were fit to double-exponential functions in KinTek Global Kinetic Explorer. The function used for fitting was *y* = *a*_0_ + *a*_1_(1 – e^−*b*1*t*^) + *a*_2_(1 – e^−*b*2*t*^), where *y* = fluorescence intensity (arbitrary units), *t* = time (s), *a*_1_ = the amplitude of the first phase, *b*_1_ = the rate of the first phase, *a*_2_ = the amplitude of the second phase, *b*_2_ = the rate of the second phase and *a*_0_ = the initial fluorescence amplitude (arbitrary units).

### Kinetic measurements by PydC fluorescence

PydC fluorescence kinetics were measured in the same buffer as in the equilibrium Trp fluorescence experiments at 22 ± 1°C using an Applied Photophysics SX.18MV stopped-flow spectrometer (Leatherhead, UK). The excitation wavelength was 350 nm, and emission was collected with a 385-nm cut-off filter. The concentrations after 1:1 mixing were 2.5, 5.0, 7.5 and 10 μM CcrM, 60.0 μM SAM and 1 μM dsDNA. Data collection and analysis was identical to the procedure used for the Trp fluorescence experiments.

### Kinetic measurements by PydC fluorescence over 2000 s

Kinetic measurements over 2000 s were performed at room temperature on a Horiba Fluoromax-4 spectrofluorometer in kinetics mode. The monochromator excitation wavelength was 350 nm with a 2 nm bandpass and the emission monochromator was parked at 450 nm with a 2 nm bandpass. The reaction was initiated with protein and hand-mixing was carefully completed in a quartz cuvette before data acquisition began. The mixing-time (10 s) was considered the deadtime for this experiment. The reaction consisted of P1-DNA [1.0 μM], SAM [60.0 μM], and enzyme [5.0 μM].

### Fluorescence anisotropy to estimate *K*_D_

Fluorescein (56-FAM) DNA oligos were obtained from Integrated DNA Technologies. DNA was annealed by melting at 95°C for 5 min followed by passive cooling to room temperature for 3 h. Native PAGE gels determined >98% purity of annealed double-stranded DNA. Binding reactions consisted of 10 nM FAM-DNA, 0–1000 nM protein, 30 μM SAH, in 50 μl volumes in the following buffer: 100 mM HEPES, 1 mM EDTA, 20 mM NaCl, pH 8. Reactions were loaded into a Corning 96-well flat-bottom black plate and incubated at room temperature for 20 min. Fluorescence anisotropy was monitored using a Tecan Spark microplate reader at room temperature. Excitation and emission polarizers were 485nm and 535nm, respectively. Anisotropy (a.u.) was plotted vs. protein concentration. Anisotropy data was background subtracted (background = FAM-DNA in buffer alone) and fit to a specific binding with Hill slope equation (Anisotropy = Anisotropy_max_*[CcrM]^*h*^/(*K*_d_^*h*^ + [CcrM]^*h*^)) using GraphPad Prism 7.00.

### Global fitting

Global data fitting was performed in KinTek Global Kinetic Explorer Version v11.0.1. The reaction scheme used as the unifying model to describe the experimental data was E + S = FS = GS^I^ = GS^II^ = GS_p_^II^ = E + S_p_. Data from four experiments were input and conditions were consistent with how the data was collected. Time-dependent data were corrected for the measured dead time of the instrument (2.6 ms) by excluding the first two data points that were collected within the first 3ms. Each experiment had a unique observable signal which relates the experimental data to the model. For example, Experiment 1 is PydC kinetics over 2 s, where PydC fluorescence depends on the following observable signal: a1*(S + FS + e*GS^I^ + f*(GS^II^ + GS_p_^II^) + h*S_p_). This observable signal describes that GS^I^ partially contributes to the change in PydC signal, while GS^II^ and GS_p_^II^ also contribute, and that GS^II^ and GS_p_^II^ contribute equally to the change in fluorescence. Experiment 4 is the Trp kinetic trace which depends on the following observable signal: a4*(E + k*FS + m*GS^I^ + n*(GS^II^ + GS_p_^II^)). Exp 3 is PydC data over a long time-course to observe the annealing of the methylated DNA strands after methylation. Experiment 3 had an observable signal identical to experiment 1 because they are both monitoring PydC signal, but over different time-bases. Experiment 3 had an observable signal of a3*(S + FS + e*GS^I^ + f*(GS^II^ + GS_p_^II^) + h*S_p_). Experiment 2 is the radiochemical methylation assay, and we therefore have an observable signal of GS_p_^II^ + S_p_ + bkg2.

Data from all four experiments were fit globally based on numerical integration of the rate constants (computer simulation). Initial values were estimated based on the fitting of each data by equation. Fluorescence scaling factors were applied to the data in experiments 1 and 4 to normalize variability in the starting amplitudes for each trace within a concentration series. Scaling factors were close to unity and therefore did not influence the concentration dependence of these traces. Some values were locked during the simulation, while others were allowed to float, as described. Locked values were chosen based on the parameter's lower limit beyond which there is no affect on the fitted curves.

Three individual models were built (WT/cognate DNA, R350A/cognate DNA, WT/Noncognate DNA and W332Y/cognate DNA) with identical model architecture for direct comparison. An alternative presentation for the R350A and Noncognate models would have been to truncate the models only showing phases that were described by the data. However, we opted for the 5-step model for a direct comparison to the WT/cognate model. Confidence contour analysis represent the 1D Fitspace calculated for each rate constant, which outlines the space over which parameters can vary. The 95% Chi^2^ limits were calculated in KinTek Explorer.

## RESULTS

### Equilibrium DNA binding

Fluorescence-based anisotropy using a 29mer hemimethylated DNA with one centrally located 5′GANTC3′ site and a 5′-FAM tag was used to estimate the apparent *K*_d_ (*K*_d_^app^). The *K*_d_^app^ for WT CcrM with cognate DNA (C1-FAM) was determined to be 150 ± 5 nM (Supplementary Fig. 2A). The *K*_d_^app^ for WT CcrM with noncognate DNA (NC-FAM) was 135 ± 8 nM (Supplementary Fig. 2B). The binding affinity for NC-FAM DNA is comparable to C1-FAM DNA suggesting that substrate binding is nonspecific and does not contribute to CcrM’s highly discriminating mechanism. DNA sequences are listed in Table 1.

**Table 1.** Names and sequences of 29mer DNA substrates. C1 is cognate DNA, P1 is cognate DNA with PydC inserted at the N-position of the recognition site, NC is noncognate DNA, PNC is noncognate DNA with PydC at the N-position of recognition site. C1-FAM is cognate DNA with a FAM-tag on the 5’ end of the target strand. NC-FAM is noncognate DNA with a FAM-tag on the 5’ end of the target strand. Underlined based identify the recognition site. P = Pyrrolo-dC, M = N6-methyl adenine, bold bases = noncognate substitutions

**DNA name and sequence**	
C1	5’-TCACTGTACTCT GACTC GCCTGACATGAC-3 ’
	3’-AGTGACATGAGA CTG M G CGGACTGTACTG-5’
P1	5’-TCACTGTACTCT GA P TC GCCTGACATGAC-3’
	3’-AGTGACATGAGA CTG M G CGGACTGTACTG-5’
NC	5’-TCACTGTACTCT GACT**G** GCCTGACATGAC-3’
	3’-AGTGACATGAGA CTG M **C** CGGACTGTACTG-5’
PNC	5’-TCACTGTACTCT GA P T**G** GCCTGACATGAC-3’
	3’-AGTGACATGAGA CTG M **C** CGGACTGTACTG-5’
C1-FAM	5’-(56-FAM)TCACTGTACTCT GACTC GCCTGACATGAC-3’
	3’-AGTGACATGAGA CTG M G CGGACTGTACTG-5’
NC-FAM	5’-(56-FAM)TCACTGTACTCT GACT**G** GCCTGACATGAC-3’
	3’-AGTGACATGAGA CTG M **C** CGGACTGTACTG-5’

### Equilibrium Trp fluorescence

**Figure 2.**
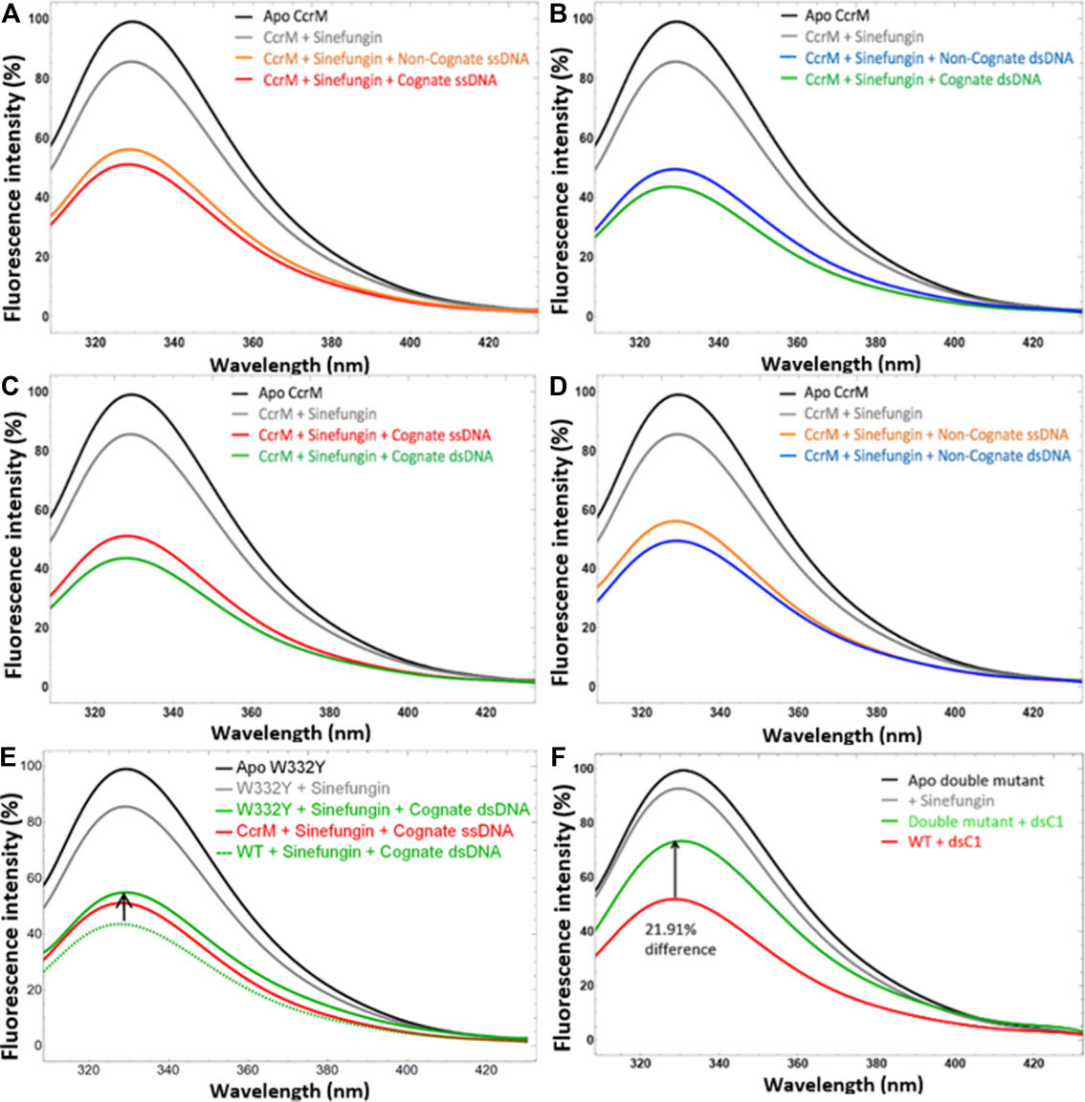
Equilibrium Trp fluorescence shows minor differences in the presence of ssDNA vs dsDNA and cognate versus noncognate DNA. Equilibrium fluorescence was measured for the binding of cognate or non-cognate DNA. (**A**) Difference in cognate vs non-cognate ssDNA. (**B**) Difference in cognate versus non-cognate dsDNA. (**C**) Difference in cognate ssDNA or dsDNA. (**D**) Difference in non-cognate ssDNA or dsDNA. Binding of ssDNA induced smaller fluorescence changes than dsDNA independent of whether the strands were cognate or non-cognate. (**E**) WT and W332Y binding to ssDNA give the same result (red). The arrow shows that the fluorescence of W332Y is only different than that of WT when bound to dsDNA. (**F**) The double variant W332Y/W335Y displays lesser relative fluorescence change than WT for binding to dsDNA, suggesting that the WT effect is due to contributions from both the N-terminus and C-terminus.

Equilibrium tryptophan fluorescence was measured upon the binding of cognate (C1) and noncognate (NC) DNA (Table 1). There are 18 Trp residues per dimer of CcrM (Figure 1A). As a result of CcrM binding to DNA, the protein fluorescence is significantly decreased (Figure 2). Consistent with our prior finding that CcrM methylates ssDNA as efficiently as dsDNA, we observe similar reductions in protein fluorescence with both ssDNA and dsDNA (Figure 2C, 30). Interestingly, we observe similar changes in protein fluorescence with noncognate DNA, suggesting nonspecific protein conformational changes occur even though noncognate DNA shows no evidence of strand separation (Figure 2A, B, 11). Therefore, the protein conformational change monitored by Trp fluorescence is not significantly influenced by the event of strand separation. Rather, as we show subsequently, Trp fluorescence monitors a protein conformational change that precedes DNA strand separation. We know from the crystal structure and previous PydC data (11) that cognate dsDNA undergoes strand separation, while non-cognate dsDNA is not strand separated. Therefore, the equilibrium Trp fluorescence occurs regardless of DNA strand separation. Binding of ssDNA induced smaller conformational changes than dsDNA, independent of whether the strands were cognate or non-cognate (Figure 2C, D).

W332 and W335 are the only two Trp residues in the C-terminal segment (Figure 1A). The DNA-dependent change in protein fluorescence observed with the W332Y/W335Y double variant is significantly attenuated compared to the WT (Figure 2F), suggesting that these residues and the C-terminal segment contribute to the fluorescence changes observed upon DNA binding. WT and W332Y binding to ssDNA give the same result (Figure 2E, red trace). However, only when bound to dsDNA is the fluorescence of W332Y different than wild type (Figure 2E, green traces). This suggests that protein conformational changes associated with dsDNA are dependent on interactions from the C-terminal domain, while changes associated with ssDNA are not dependent on such interactions.

### Kinetic model for WT and cognate DNA

**Figure 3.**
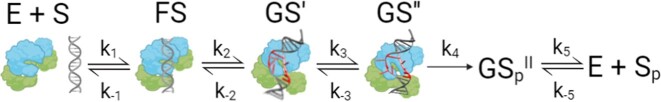
Conformational changes in the protein and DNA that lead up to methylation by WT CcrM. Enzyme conformational states detected by changes in Trp fluorescence are designated as *E*, *F* and *G*. DNA strand-separated states detected by PydC fluorescence are designated as *S* (duplex), *S*^I^ (partially strand-separated intermediate), and *S^II^* (fully strand-separated). *Sp* designates the methylated DNA product. In step 2, the protein isomerization and the first DNA strand-separation step appear to be coincident. SAM is not included in the kinetic scheme because SAM is in excess in all experiments, therefore SAM is assumed to be bound to all enzyme species.

**Figure 4.**
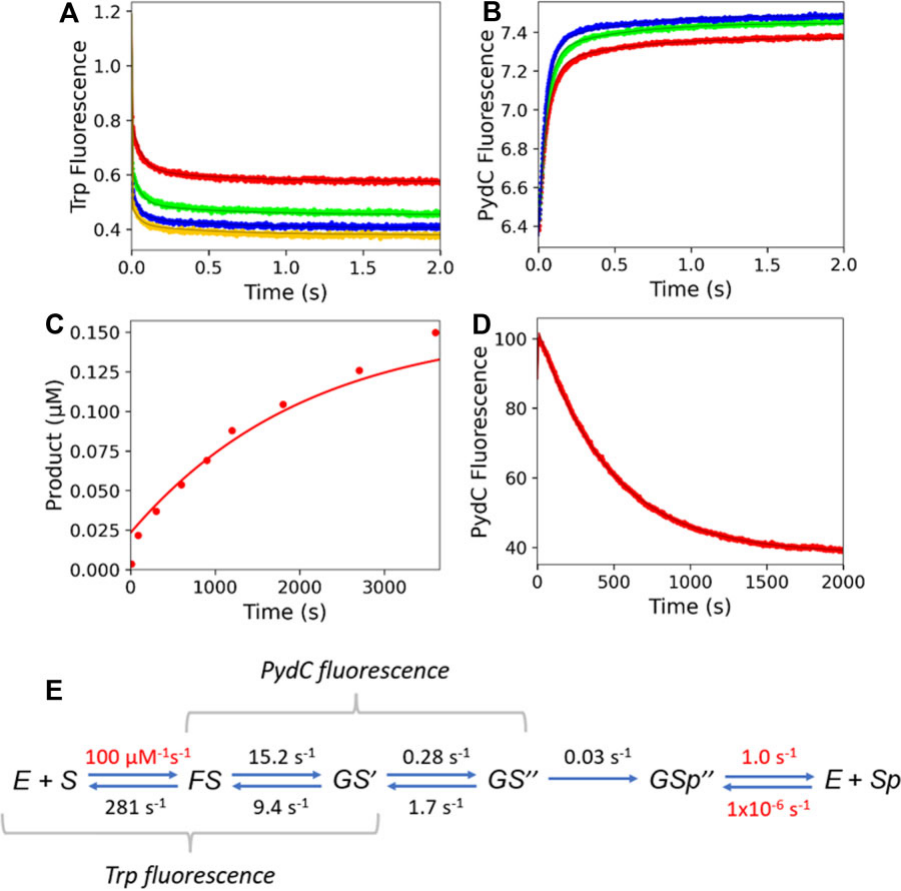
Global fitting of the data defining the kinetic steps for WT CcrM and Cognate DNA. (**A**) Trp kinetics consisted of WT CcrM [500 nM], C1-DNA [2.5 (red), 5.0 (green), 7.5 (blue) and 10.0 (yellow) μM], and SAM [60 μM]. (**B**) DNA strand-separation kinetics monitored by PydC fluorescence consisted of P1-DNA [1.0 μM], WT CcrM [5.0 (red), 7.5 (green) and 10.0 (blue) μM], and SAM [60 μM]. (**C**) WT and cognate DNA single turnover methylation monitored via radiochemical methods. P1-DNA [100 nM], WT [250 nM], and SAM [15 μM]. (**D**) PydC kinetics over 2000 s monitored product DNA annealing and/or release, and consisted of WT [5 μM], DNA [1 μM] and SAM [60 μM]. All experiments in each model were globally fit in KinTek Explorer. Solid lines represent the simulated traces from the global fit. (**E**) Rate constants derived from globally fitting the data for WT and Cognate DNA. Red values indicate rates that were locked during the simulation.

As detailed below, our data support a kinetic model to describe the conformational changes in the protein and DNA that lead up to methylation by CcrM (Figure 3). The model was derived to account for data from four experiments: Trp fluorescence, PydC fluorescence over 2 s, PydC fluorescence over 2000 s, and a radiochemical single-turnover methylation assay. Global data fitting of experiments performed with WT CcrM and cognate DNA defined the kinetically significant steps leading to DNA methylation as shown in Figure 4. Enzyme conformational states detected by changes in Trp fluorescence are designated as *E*, *F* and *G*. DNA strand-separated states detected by PydC fluorescence are designated as *S* (duplex), *S*^I^ (partially strand-separated intermediate), and *S^II^* (fully strand-separated). *Sp* designates the methylated DNA product. In step 2, the protein isomerization and the first DNA strand-separation step appear to be coincident.

### Kinetics for WT and cognate DNA

Trp fluorescence is biphasic, and the fast phase (*k*_1_) is concentration-dependent and represents the association of CcrM and DNA. The second slower phase of Trp fluorescence (*k*_2_) monitors an isomerization event in the protein after DNA binding (Figure 4A). The first fast phase (*k*_1_) is evident at the lowest DNA concentration, but lost in the dead time of the stopped flow at the higher DNA concentrations. The fluorescence amplitude of the first Trp phase decreases with increasing enzyme, suggesting that at higher enzyme concentrations, more FS complex has formed during the deadtime of the experiment (Supplementary Figure 3A, B). The deadtime of the stopped-flow is approximately 2.6 ms, therefore two data points within the first 3ms were excluded from each trace (Supplementary Figure 1).

Global fitting reveals that when the Trp data is extrapolated back to zero time, there is a concentration-dependent loss of signal amplitude. Global data fitting accounting for the observed signal and loss of amplitude in the fast phase provides a minimal estimate of the DNA binding rate constant of approximately 100 μM^−1^ s^−1^. During global data fitting *k*_1_ is locked because it is not well-defined. Accordingly, fitting to derive *k*_–1_ affords an estimate of the *K*_d_ for formation of the initial DNA-bound state. Importantly, the data describe two changes in tryptophan fluorescence: one occurring simultaneously with DNA binding and a second change in fluorescence occurring after DNA binding.

Incorporation of PydC in place of N in the target strand (5′GANTC3′) provides a strong signal for strand separation as supported by extensive prior controls (11). As is often the case in analog studies, this alters the enzyme activity (k_methylation_) (Supplementary Figure 9). The apparent *k*_methylation_ with C1-DNA is 1.4 min^−1^ which is decreased with P1-DNA to 0.05 min^−1^, which may not perfectly reflect the rate constants with the unlabeled duplexes. However, the prior controls and those included here provide strong support for the relevance of using PydC in our studies.

PydC fluorescence monitoring DNA strand-separation is also biphasic with a fast change in fluorescence followed by a slower signal with a lower amplitude (Figure 4B). The first phase of PydC fluorescence and the second phase of Trp fluorescence appear to be coincident, therefore we have defined this coincident step as *k*_2_. In the WT/CognateDNA model, GS^I^ represents an intermediate in which both the protein has undergone an isomerization event and the DNA has been partially strand-separated. The second phase of PydC kinetics is the second phase of DNA strand separation which we have defined as k_3_. The apparent rates of these two phases are independent of concentration over the range tested, indicating that DNA strand-separation is a first-order process (Supplementary Figure 3C, D).

On a longer timescale (Figure 4D) the PydC signal decreases as the product DNA is released from the enzyme and re-anneals to form duplex, although we cannot define the order of these two steps. The data can be fit with fast product release by assigning *k*_5_ = 1 s^−1^ so it is greater than k_4_. Accordingly, the decrease in the PydC signal on the long timescale defines the rate of DNA methylation (Figure 4E) consistent with the direct measurement of DNA methylation in a single-turnover using radiochemical methods (Figure 4C).

Globally fitting these data show that the DNA binding is fast and reaches equilibrium as measured by the fast tryptophan fluorescence change. The second change in tryptophan fluorescence indicating a change in enzyme structure is correlated with the first DNA strand-separation step to form an unknown intermediate state, preceding to a second change in PydC signal that is then followed by DNA methylation (Figure 4E).

### Kinetic model for WT and noncognate DNA

**Figure 5.**
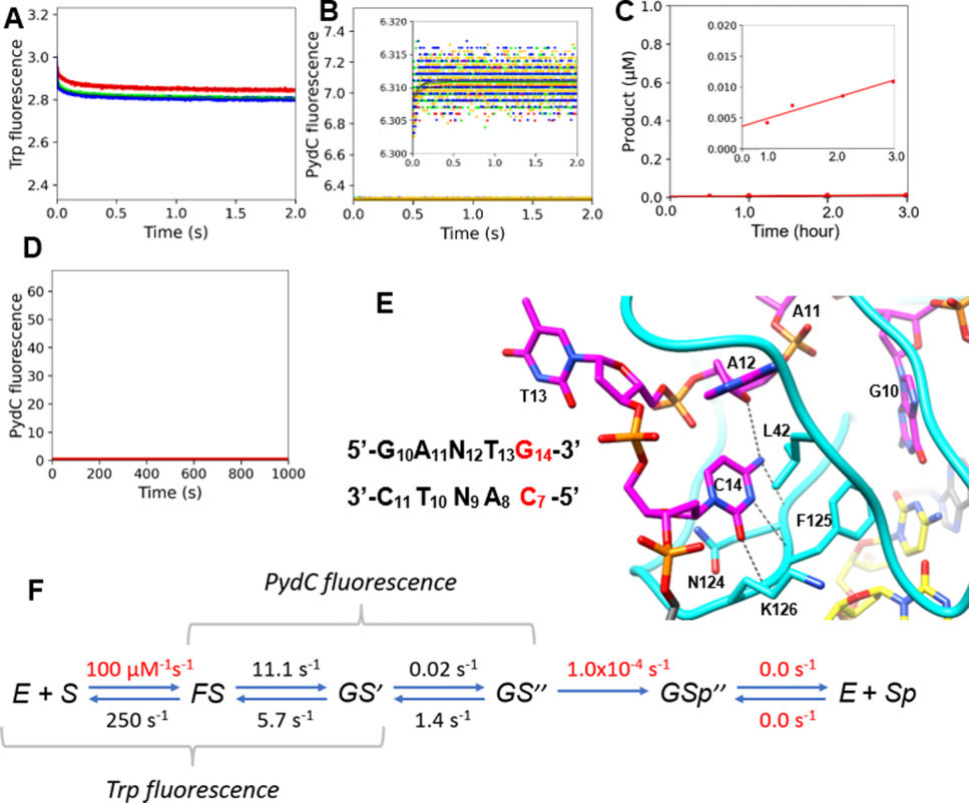
WT and Noncognate DNA Trp kinetics are only slightly perturbed while PydC kinetics are not observed, suggesting that DNA strand-separation is tightly coupled to substrate discrimination. (**A**) Trp kinetics consisted of WT [500 nM], NC-DNA [2.5 (red), 7.5 (green) and 10.0 (blue) μM], and SAM [60 μM]. (**B**) DNA strand-separation monitored by PydC fluorescence consisted of PNC-DNA [1.0 μM], WT [2.5 (red), 5.0 (green), 7.5 (blue) and 10.0 (yellow) μM], and SAM [60 μM]. (**B**, inset**)** Increased resolution in the y-axis of (B). (**C**) WT and noncognate DNA single turnover methylation assay. An important distinction in the conditions for the noncognate methylation assay; enzyme and DNA concentrations were increased to bring the radiochemical signal above baseline levels and the reaction went longer. NC-DNA [1.0 μM], WT [2.5 μM], SAM [15 μM]. (**C**, inset) Increased resolution in the *y*-axis of (C). (**D**) PydC kinetics over 1000 s consisted of WT [5 μM], NC-DNA [1 μM] and SAM [60 μM]. (**E**) The noncognate DNA has one base pair substitution in the recognition site. Cytosine14 is replaced with guanine on the target strand and G7 is replaced with cytosine on the nontarget strand. The red bases represent the noncognate substitution. Cytosine14 makes 3 hydrogen bonds from its base to the peptide backbone of K126, F125 and N124 and to the pentose ring of A12. C14 appears to be stacked between N124 and L42. (**F**) Rate constants derived from globally fitting the data for WT and Noncognate DNA. Red values indicate rates that were locked during the simulation.

Figure 5 shows the global fitting of data describing the reaction of WT CcrM and noncognate DNA (5′-GANTG-3′), which has one basepair changed from the cognate sequence. For direct comparison with cognate DNA, we fit the data globally using the same model as was derived for cognate DNA, although the steps and rate constants were not well resolved.

Trp kinetics with WT and noncognate DNA are similar to that observed with cognate DNA (Figure 5A) but with a decreased amplitude in both phases (Supplementary Figure 4A, B). PydC fluorescence with noncognate DNA shows no change indicating that noncognate DNA does not undergo DNA strand separation (Figure 5B, Supplementary Figure 4C, D). The most obvious results shown in Figure 5 are that DNA strand-separation does not occur (Figure 5B), the rate of DNA methylation is reduced at least 300-fold (Figure 5C), and subsequent product annealing and release is not observed (Figure 5D). Thus, although the second tryptophan fluorescence change appears to be coincident with the first DNA strand-separation step for cognate DNA, these data demonstrate that the two events are not coupled.

The noncognate DNA has cytosine14 replaced with guanine on the target strand and G7 is replaced with cytosine on the nontarget strand (Figure 5E). Cognate Cytosine-14 makes 3 hydrogen bonds from its base to the peptide backbone of K126, F125, and N124 and to the pentose ring of A12. Moreover, C14 appears to be stacked between N124 and L42 (Figure 5E).

The WT/Noncognate DNA model reveals that the substrate discriminating step is DNA strand separation. The equilibrium constant *K*_1_ is slightly greater with noncognate DNA implying that CcrM binds noncognate DNA tighter than cognate DNA. This is consistent with the apparent *K*_d_ estimated from anisotropy data (Supplementary Figure 2) and prior Kd estimates via EMSA methods (22). The equilibrium constant *K*_2_ in the noncognate model favors the forward progression in the pathway slightly more than *K*_2_ in the cognate model, but for noncognate DNA, this step is defined solely by the Trp fluorescence signal. Although global data fitting derives an estimate for *k*_3_, this is based on a small signal that is comparable to the noise in the data (Figure 5B) and is therefore not deemed to be reliable.

The lack of an observable PydC signal indicates that GS^I^ does not progress to GS^II^ with noncognate DNA, which is consistent with the low level of methylation activity (Figure 5F), which is at least 300-fold slower than with cognate DNA.

### Kinetic model for R350A and cognate DNA

**Figure 6.**
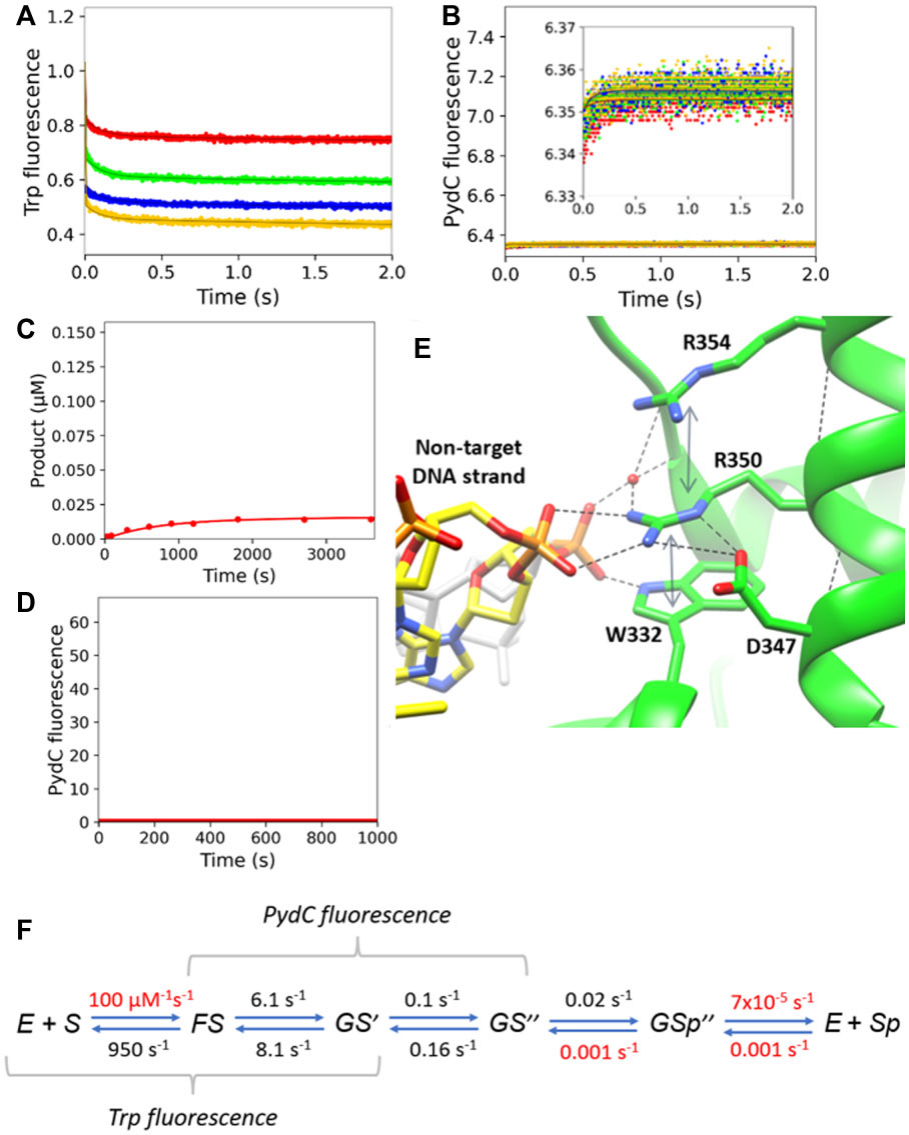
R350A has severely attenuated PydC fluorescence while Trp kinetics are only slightly perturbed, suggesting that the protein isomerization step and DNA strand separation are mechanistically uncoupled. (**A**) Trp kinetics consisted of R350A [500 nM], C1-DNA [2.5 (red), 5.0 (green), 7.5 (blue) and 10.0 (yellow) μM], and SAM [60 μM]. (**B**) DNA strand-separation monitored by PydC fluorescence consisted of P1-DNA [1.0 μM], R350A [5.0 (red), 7.5 (green) and 10.0 (blue) μM], and SAM [60 μM]. (**C**) R350A and cognate DNA single turnover methylation monitored via radiochemical methods. P1-DNA [100 nM], R350A [250 nM] and SAM [15 μM]. (**D**) PydC kinetics over 1000 s consisted of R350A [5 μM], P1-DNA [1 μM] and SAM [60 μM]. (**E**) R350 from the C-terminal domain makes two hydrogen bonds to the phosphate backbone of the non-target strand (yellow DNA). Hydrogen bonds are black dashed lines. Stacking interactions are depicted with grey arrows. Molecular graphics were made with UCSF Chimera. (**F**) Rate constants derived from globally fitting the data for WT and Noncognate DNA. Red values indicate rates that were locked during the simulation.

R350A displays Trp kinetics similar to WT, while not displaying a change in PydC signal (Figure 6A, B, Supplementary Figure 5B–D), providing further evidence that the protein isomerization and DNA strand separation are uncoupled. The protein fluorescence change still reflects fast DNA binding and a possible change in enzyme conformation occurring in two steps. R350A displays biphasic Trp kinetics with apparent rates slightly less than WT, and the fluorescence amplitudes in both phases are slightly reduced (Figure 6A, Supplementary Figure 5A, B). R350A displays severely attenuated PydC signal (Figure 6B).

Surprisingly, the methylation by R350A still proceeds as a rate comparable to WT, but the amplitude is reduced to approximately 15% of the DNA forming product (Figure 6C) in this single turnover experiment. This mutant reveals unexpected behavior in that less than the expected amount of product is generated (only 15%) and DNA strand-separation is negligible. The mutant may allow a fraction of the adenine in the target strand to reach the active site without DNA strand-separation. The parameter *k*_-4_ was proposed in the R350A/CognateDNA model to account for the incomplete methylation reaction by allowing the chemistry step to come to equilibrium with slow product release. Thus, the single-turnover methylation assay does not go to completion (Figure 6C), and subsequent product annealing and release is not observed (Figure 6D). Alternatively, R350A could have an off-pathway intermediate in which we did not incorporate into the model. However, we opted for the 5-step model to keep the models consistent. Further studies are needed to define the origin of this phenomenon.

R350 from the C-terminal domain makes two hydrogen bonds to the phosphate backbone of the non-target strand (Figure 6E). Substitution of R350 with alanine disables these hydrogen bonds, resulting in destabilization of both FS and GS^I^ preventing formation of GS^II^ (Figure 6F). The functional consequences for R350A primarily are due to the increase in the rate constant k_-1_ and the decrease in the equilibrium constant *K*_2_. The equilibrium constant K2 favors the reverse of DNA binding for R350A, while WT favors going forward in the pathway. This explains the lack of PydC signal observed for R350A. Global fitting resolved a rate of DNA strand separation to be 3.5-fold slower for R350A (Figure 6F). Therefore, if GS^I^ is formed, R350A is still able to DNA strand-separate. However, the lack of PydC signal is due to the minimal population of GS^I^. R350A can methylate DNA, however product turnover is limited by the unfavorable formation of the strand-separated state (GS^II^).

### Calculations of *k*_cat_, *K*_M_ and *k*_cat_/*K*_M_

Values for *k*_cat_, *K*_M_ and *k*_cat_/*K*_M_ were calculated for each model based on equations 1, 2 and 3, respectively. These values are listed in Table 2. The specificity constant is defined by *k*_cat_/*K*_M_. These values show that the decreased specificity constant for R350A is mostly due to an increase in *K*_M_, while the larger decrease for noncognate is mostly due to a decreased *k*_cat_. Because the binding steps are much faster than DNA methylation, the *K*_m_ reflects the equilibrium binding of DNA including isomerization steps and DNA strand separation, which are unfavorable thermodynamically so only one third of the enzyme-DNA complexes reach the fully strand-separated state required for catalysis.


(1) \begin{eqnarray*} {{\mathrm{k}}}_{{\mathrm{cat}}} = \frac{{{{\mathrm{k}}}_2{{\mathrm{k}}}_3{{\mathrm{k}}}_4}}{{{{\mathrm{k}}}_2({{\mathrm{k}}}_3 + {{\mathrm{k}}}_{ - 3} + {{\mathrm{k}}}_4) + {{\mathrm{k}}}_{ - 2}({{\mathrm{k}}}_{ - 3} + {{\mathrm{k}}}_{ - 4}{\mathrm{) + }}{{\mathrm{k}}}_3{{\mathrm{k}}}_4}} \nonumber \\ \end{eqnarray*}

(2) \begin{eqnarray*}{{\mathrm{K}}}_{\mathrm{M}} = \frac{{{{\mathrm{k}}}_{ - 1}{\mathrm{(}}{{\mathrm{k}}}_{ - 2}{{\mathrm{k}}}_{ - 3}{\mathrm{ + }}{{\mathrm{k}}}_{ - 2}{{\mathrm{k}}}_4{\mathrm{ + }}{{\mathrm{k}}}_3{{\mathrm{k}}}_4{\mathrm{) + }}{{\mathrm{k}}}_2{{\mathrm{k}}}_3{{\mathrm{k}}}_4}}{{{{\mathrm{k}}}_1[{{\mathrm{k}}}_2({{\mathrm{k}}}_3 + {{\mathrm{k}}}_{ - 3} + {{\mathrm{k}}}_4){{\mathrm{k}}}_{ - 2}({{\mathrm{k}}}_{ - 3} + {{\mathrm{k}}}_4{\mathrm{) + }}{{\mathrm{k}}}_3{{\mathrm{k}}}_4]}} \nonumber \\ \end{eqnarray*}

(3) \begin{eqnarray*}{{\mathrm{k}}}_{{\mathrm{cat}}}/{{\mathrm{K}}}_{\mathrm{M}} = \frac{{{{\mathrm{k}}}_1{{\mathrm{k}}}_2{{\mathrm{k}}}_3{{\mathrm{k}}}_4}}{{{{\mathrm{k}}}_{ - 1}({{\mathrm{k}}}_{ - 2}{{\mathrm{k}}}_{ - 3} + {{\mathrm{k}}}_{ - 2}{{\mathrm{k}}}_4 + {{\mathrm{k}}}_3{{\mathrm{k}}}_4) + {{\mathrm{k}}}_2{{\mathrm{k}}}_3{{\mathrm{k}}}_4}} \nonumber \\ \end{eqnarray*}

**Table 2.** *k*_cat_, *K*_M_ and *k*_cat_/*K*_M_ were calculated for each model. *k*_cat_, *K*_M_ and *k*_cat_/*K*_M_ were calculated from equations (1), (2) and (3), respectively. *k*_cat_/*K*_M_ is the specificity constant. An asterisk indicates a value that is not well defined. *k*_cat_ for R350A does not include the parameter *k*_–4_ which is greater than zero, therefore *k*_cat_ for R350A is not well-defined

	**Global fitting model**		
**Parameter**	**WT/Cognate DNA**	**R350A/Cognate DNA**	**WT/Noncognate DNA**
*k*_cat_ (s^−1^)	0.0027	*0.0039	1.0 × 10^−6^
*K*_M_ (μM^−1^)	0.99	4.39	0.85
*k*_cat_/*K*_M_ (μM^−1^ s^−1^)	0.0027	0.00088	1.2 × 10^−6^

### Confidence contour analysis

**Figure 7.**
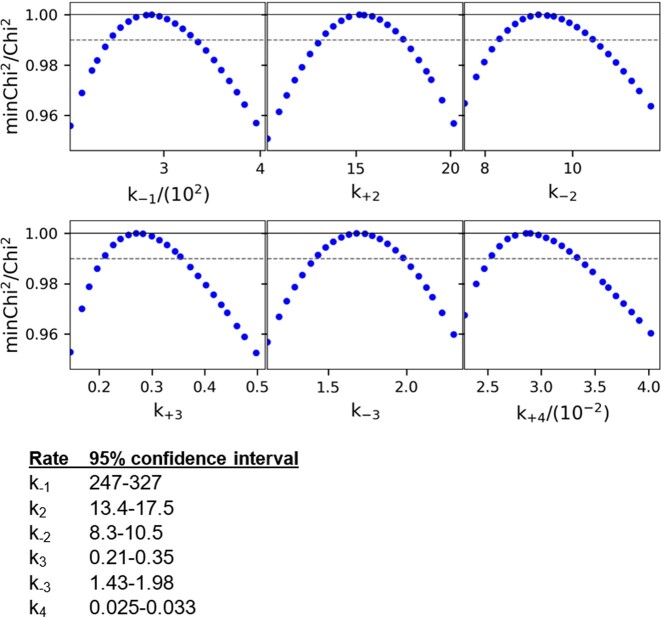
Confidence contour analysis show that the data are well-defined for the WT and cognate DNA model. The data represent the 1D Fitspace calculated for each rate constant, which outlines the space over which parameters can vary. The dashed line establishes the 95% confidence interval at the 0.99 Chi^2^ threshold. The 95% Chi^2^ limits were calculated in KinTek Explorer and are shown in the table.

Confidence contour analysis show that the kinetic parameters are well-defined for the five step model with WT enzyme with cognate DNA (Figure 7). The data in Figure 7 represent the 1D FitSpace calculated for each rate constant, which outlines the space over which parameters can vary. The dashed line establishes the 95% confidence interval at the 0.99 normalized Chi^2^ threshold. The 95% Chi^2^ limits were calculated in KinTek Explorer and are shown in Figure 7. The confidence contour 1D FitSpace for the WT/Noncognate DNA and R350A/CognateDNA models are shown in Supplementary Figures 6 and 7, respectively.

### Kinetics of PydC in the absence of cofactor

**Figure 8.**
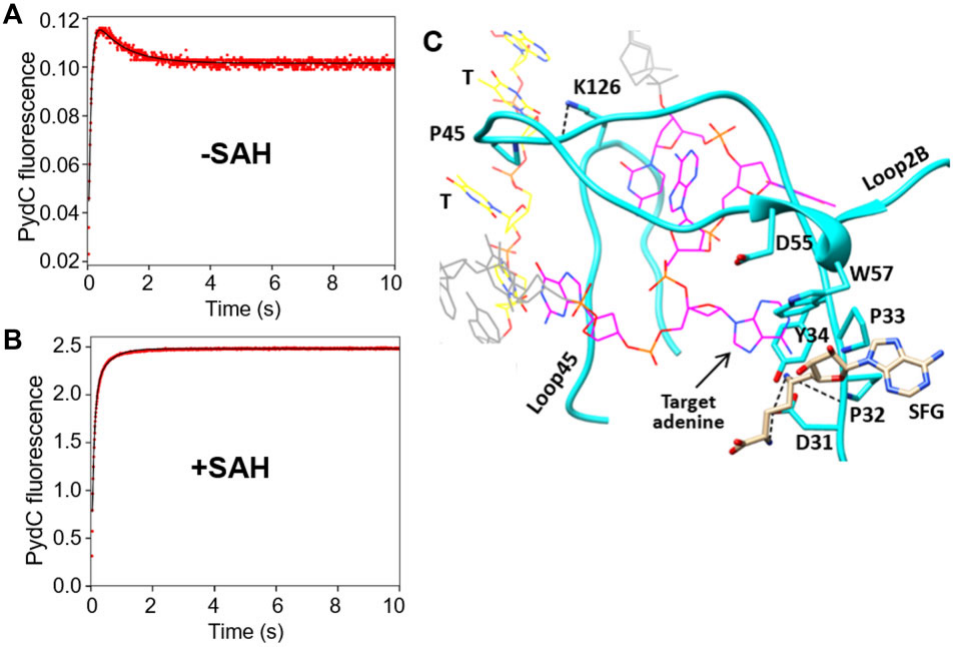
PydC kinetics in the absence of cofactor suggest that the cofactor stabilizes the strand-separated DNA conformation (GS^II^). (**A**) We observe biphasic PydC kinetics for WT CcrM in the absence of SAH. When fit to a double-exponential function, the initial increase in fluorescence has an apparent rate of 11.4 s^−1^ with an amplitude of 0.14, and the second decreasing phase has an apparent rate of 1.07 s^−1^ with an amplitude of 0.02. (**B**) In the presence of SAH, we do not observe the reversal of the signal. When fit to a double-exponential function, the fast initial increase in fluorescence has an apparent rate of 13.3 s^−1^ with an amplitude of 2.4 and the second slower phase has an apparent rate of 2.3 s^−1^ with a fluorescence amplitude of 0.5. The conditions consisted of WT CcrM [5 μM], P1-DNA [1 μM] and SAH [0 or 60 μM]. (**C**) The crystal structure suggests that SAH stabilizes the strand-separated conformation (GS^II^) as seen by interactions between the analog sinefungin (SFG) and the DPPY (D31-Y34) motif in Loop2B. Magenta and yellow DNA represent the recognition site of the target and non-target strands, respectively, while grey DNA is outside of the recognition site. Sinefungin (tan) makes hydrogen bonds (black dashed lines) to D31 and the peptide backbone of P32. W57 makes a stacking interaction to the dihydroxyoxolan of sinefungin. P45 is intercalated between two thymine bases of the non-target DNA strand (yellow). Molecular graphics were made with UCSF Chimera.

In order to understand the role that cofactor plays in DNA strand separation we compared the changes in fluorescence of the PydC signal in the presence and absence of the cofactor. In the absence of cofactor, PydC signal is 5-fold smaller than in the presence of SAH (the product of the reaction with SAM) (Figure 8A). When fit to a double-exponential function, the initial increasing phase has an apparent rate of 11.4 s^−1^ with a fluorescence amplitude of 0.14, and the second decreasing phase has an apparent rate of 1.07 s^−1^ with a fluorescence amplitude of 0.02 (Figure 8A). In the presence of SAH, (Figure 8B) the PydC signal follows a double-exponential function with a fast initial phase at an apparent rate of 13.3 s^−1^ with an amplitude of 2.4 and the second slower phase has an apparent rate of 2.3 s^−1^ with an amplitude of 0.5 (Figure 8B). While we do not understand the mechanistic basis for the small increase then decrease in fluorescence of PydC in the absence of cofactor, it is clear that a much larger signal for DNA strand separation occurs with the cofactor analog.

The similar rate of the first increasing phase with and without SAH could suggest that some strand separation can occur independent of cofactor, but the approximately 5-fold increase in the amplitude with SAH implies a much greater fraction of the DNA reaches the strand-separated state (GS^II^). Because the steps leading up to GS^II^ reach equilibrium before DNA methylation, interactions between the cofactor, enzyme and DNA will affect the fraction of bound DNA in the GS^II^ state and the observed rate for the PydC signal will be the sum of rate constant for the forward and reverse reactions (39). Our data suggest that cofactor (SAH) stabilizes the strand-separated conformation (GS^II^) leading to a much larger amplitude in the PydC signal.

The cocrystal structure relies on sinefungin (a nonreactive structural analog of SAM) to stabilize the GS^II^ complex and shows that the cofactor stabilizes GS^II^ with interactions involving loop residues. For example, W57 at one terminus of Loop2B makes a stacking interaction to sinefungin, and the catalytic DPPY motif at the other terminus of the loop makes hydrogen bonds to sinefungin (Figure 8C) (31). P45 at the proximal end of Loop2B is intercalated between two thymine bases of the nontarget strand and the peptide backbone of Loop2B makes an intra-loop hydrogen bond to the sidechain of K126 of Loop45 (Figure 8C). Therefore, when cofactor is bound, Loop2B is positioned between the separated strands of DNA and makes interactions that stabilize the strand-separated conformation (GS^II^).

**Figure 9.**
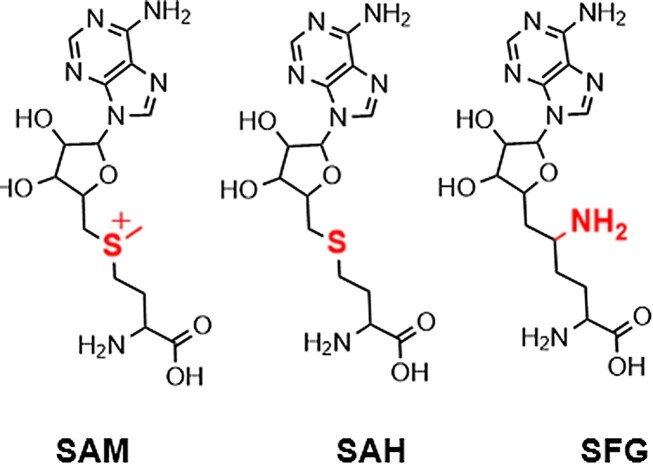
Structures of SAM, SAH, and SFG. The structure of SAM and analogs SAH and SFG are shown with the structural differences colored red. SAM is the cognate substrate and SAH is the cognate product of the methylation reaction. The analog SFG’s internal amino group is protonated, and the resulting positive charge increases its resemblance to SAM. Structural images were made with ChemDraw.

It is important to note which cofactor analog was used in the experiments throughout this work; equilibrium fluorescence measurements required a nonreactive analog (SFG) to stabilize the pre-catalytic conformation, while kinetic fluorescence measurements were able to use the reactive and cognate cofactor, SAM. The structures of each cofactor are shown in Figure 9. SFG was used in equilibrium Trp fluorescence (Figure 2) as well as the crystal structure (23). SAM was used in all experiments included in global fitting (Figures 4–6) and SAH was used to compare PydC kinetics in the absence of cofactor (Figure 8).

## DISCUSSION

How proteins bind to specific DNA sequences is well understood and sequence recognition by DNA endonucleases and methyltransferases has been extensively studied (40,41). For example, DNA methyltransferases gain stereochemical access to the target adenine or cytosine within a recognition sequence by ‘base-flipping’ of the target base (42,43–47). Conformational transitions such as base-flipping can be rate-limiting and contribute significantly to sequence discrimination (42). CcrM stands out in this context for several reasons. Its sequence discrimination is orders of magnitude more stringent than previously reported DNA methyltransferases, perhaps reflecting selective pressures associated with its essential role in gene regulation in *C. crescentus* (7,10,22). Additionally, rather than causing a single base to undergo a base-flipping transition, CcrM induces the extrahelical positioning of four of the five bases in its recognition sequence. Data presented here support our working hypothesis that this unprecedented repositioning of multiple bases into distinct recognition pockets on the protein is a major determinant of specificity. The assays and concepts being presented here for CcrM have potential relevance to other enzymes using a strand displacement recognition mechanism (e.g. CRISPR-Cas9) (3, (4) or enzymes that recognize and modify a single strand within DNA lesions or mismatches (e.g. Human MettL3-L14) (48).

### Relationship between DNA strand separation and methylation

Product release is often rate-limiting for DNA methyltransferases (49–51), but not always (52) and product release is not rate-limiting for CcrM (22,30). Our data and model (Figures 3 and 4) support this conclusion. To define the rate-limiting and specificity-determining steps we measured the rate constants of the steps leading up to the first kinetically irreversible step (methylation), including DNA strand separation. Our proposed kinetic model (Figure 3) describes a precatalytic protein conformational change followed by reversible DNA strand separation. The strand-separated conformation is the precatalytic intermediate where the target adenine is positioned for catalysis (GS^II^) as observed in the cocrystal structure depicting GS^II^ where four of the five bases of the target sequence are disrupted (Figure 1). The rates of DNA strand separation (*k*_2_ and *k*_3_) are an order of magnitude faster than methylation and they are readily reversible with both *k*_–2_ and *k*_–3_ faster than *k*_4_. DNA methylation (*k*_4_) is the rate-determining step and the actual rate is attenuated by the fact that only 10% of the enzyme-bound DNA is in the fully strand-separated state poised for catalysis (fraction = *K*_2_*K*_3_/(1 + *K*_2_ + *K*_2_*K*_3_)). The thermodynamically unfavorable strand separation may contribute to the enzyme's high fidelity in that any weakening of the interactions of the DNA with the enzyme would greatly reduce the amount of strand-separated DNA. In contrast, if the reaction went to 99% completion, a small change in free energy of strand separation due to noncognate DNA may still allow 90% strand separation. This phenomenon is similar to the weak binding of catalytic Mg^2+^ to DNA polymerases which occurs only after the induced-fit recognition of a canonical base-pair (37). Tighter binding of the catalytic Mg^2+^ would provide additional binding energy to stabilize and incorporate a mismatch.

### Relationship between protein and DNA conformational changes

We sought to monitor the steps leading up to GS^II^. We monitored equilibrium Trp fluorescence changes upon binding of different DNA substrates (Figure 2) which corresponds to the transition from E to GS^I^. Because our previous work showed, surprisingly, that CcrM methylates ssDNA and dsDNA with similar efficiency (22, (30), we showed that both cognate and non-cognate ssDNA induce the same changes in Trp fluorescence of CcrM (Figure 2A). The same result was observed with cognate and non-cognate dsDNA (Figure 2B). Together, these results suggest that protein conformational changes induced by binding DNA are not inherently dependent on the form nor sequence of the substrate. Figure 2E shows that WT and W332Y, which directly contact the non-target DNA strand (Figure 4C), have similar Trp fluorescence changes upon binding ssDNA, whereas this is not observed upon binding dsDNA by the mutant. This suggests the fluorescence changes deriving from W332 make a significant contribution to the changes observed upon binding dsDNA, and implicates the C-terminal domain's importance in these changes.

We utilized stopped-flow fluorescence to quantify the rates of the changes in protein conformation and DNA strand separation. Our model suggests that a protein conformational change occurs after the association of enzyme and DNA, followed by DNA strand-separation, generating the intermediate poised for methylation (GS^II^). The apparent rates of Trp and PydC kinetics are effectively identical for WT CcrM with cognate DNA (C1) (Figure 4A, B). Our interpretation of the coincident rates is that the protein conformational change precedes and limits the rate of the first DNA strand-separation step to form the intermediate state (GS^I^). Thus, the first change in PydC fluorescence is actually reflecting the rate of the protein conformational change.

### Relationship between DNA strand separation and target sequence recognition

We suggest that DNA discrimination is dependent on strand-separation, while protein conformational changes do not contribute significantly to DNA sequence discrimination. Similar Trp kinetics are observed with both cognate and noncognate DNA, suggesting that CcrM undergoes similar protein conformational changes with both substrates (Figure 5A compared to Figure 4A). Also, PydC kinetics are not observed with noncognate DNA (Figure 5B). The inability to strand-separate noncognate DNA while the protein conformational change is unperturbed, provides additional evidence that these processes are independent. The interactions between features of CcrM and either the target or non-target DNA strands observed in the cocrystal structure, suggest potential roles in inducing or stabilizing the strand separated intermediate (GS^II^, Figure 2B). For example, three loops (2B, 45, and 6E) interact with specific bases of the target strand 5′GANTC′3 site (23). Loop2B and Loop45 are inserted between the strand-separated DNA, suggesting they contribute to strand separation or maintain the strand separated form (GS^II^). Loop6E may contribute to the stabilization of GS^II^ and recognition of specific nucleic acid bases since it is not positioned between the strand-separated DNA and it makes base-specific interactions with the recognition site.

Noncognate DNA is able to bind to the enzyme, but the DNA strand-separated state is destabilized and does not accumulate, leading to reduced methylation. This observation supports the conclusion from studies on cognate DNA that discrimination against noncognate DNA is facilitated by equilibria that disfavor DNA strand-separation. Thus, a small change in equilibrium constant for stabilizing the strand-separated state at the active site translates into a large factor of discrimination against DNA methylation of noncognate DNA. Note that the rate constants governing steps 1 and 2 with noncognate DNA were derived solely from the tryptophan fluorescence data while steps 3 and 4 are not well defined because their amplitudes are so small.

Our data analysis relied on global fitting which is based on numerical integration of rate equations from multiple experiments (39,53). Global fitting reveals that DNA strand separation is the substrate discriminating step. We explored a model that included an additional and much faster rate of DNA strand separation (200–300s^−1^); however, the fast phase was not described kinetically by the data. This taught us an important lesson: when deciding how many phases to include in global fitting, only steps that are described by the data may be included in defining a minimal model. The additional step may occur but it is not defined by the data. If there is a fast phase of DNA strand separation, it is not resolved with PydC.

CRISPR/Cas9 also relies on a DNA strand displacement mechanism for target DNA recognition and access (54). Unlike CcrM, CRISPR/Cas9 is not highly discriminating against off-target DNA, and once bound to an off-target site Cas9 performs DNA cleavage unless the rate of hydrolysis is exceedingly slow (54,55). CcrM’s discrimination, however, is tightly coupled to the strand-separation event, and methylation will not occur on non-cognate sites due to its highly selective DNA strand separation and recognition mechanism. Similarly to CcrM, however, CRISPR-Cas9 recognition and cleavage depends on the conformation of the guide RNA-DNA duplex (54). CRISPR-Cas9 favors cleavage of a kinked cognate duplex, and favors release of a linear mismatched duplex (55). The design of CRISPR/Cas9 variants with higher selectivity is limited by the stability of the RNA/DNA duplex. This contrast further illuminates the role DNA strand-separation can play in sequence fidelity.

CcrM’s mechanism of discrimination can also be compared to that of T7 DNA polymerase, in which a conformational change in the protein that is much faster than chemistry selects the correct nucleotide via an induced-fit mechanism (34). Similarly, CcrM’s discriminating strand-separation step is faster than the methyl-transfer chemistry. However, in the case of DNA polymerase, dissociation of the bound nucleotide is modulated so that a correct base pair is captured and committed to incorporation, while a mismatch is rapidly released rather than incorporated. In the case of CcrM the strand-separated state with noncognate DNA is so disfavored that it cannot be observed. Presumably, this is due primarily to a fast dissociation rate that we cannot measure because we can’t form the bound strand-separated state with noncognate DNA. The arguments are similar to the controversies over the role of induced-fit in specificity where substrate-induced changes in enzyme structure were thought to be unimportant for specificity unless they were rate-limiting (33). Here, we show that the rate of DNA strand-separation is much faster than DNA methylation, but still constitutes a major determinant of specificity.

This model tells a story where DNA strand-separation is 100-fold faster than DNA methylation which does not occur until the DNA is fully strand-separated. However, DNA strand-separation is not thermodynamically favorable; rather, the unwinding comes to equilibrium with a net equilibrium constant defined by *K*_2_*K*_3_ = 0.27. The fraction of DNA in the fully strand-separated state is *K*_2_*K*_3_/(1 + *K*_2_ + *K*_2_*K*_3_) = 0.09. Thus, DNA strand-separation does not drive the reaction forward toward the reactive state. Rather, a small fraction of DNA is unwound and aligned for catalysis. There may be additional steps leading to alignment of the adenine, SAM and catalytic residues that may limit the net rate of methylation. Because DNA binding to the enzyme does not drive DNA strand-separation, discrimination against noncognate DNA may occur by allowing DNA release from any bound state prior to catalysis.

Our results with the R350A CcrM mutant support our proposed separation of protein conformational changes and DNA strand separation (Figure 6). R350 is highly conserved in the C-terminal domain and makes two hydrogen bonds to the phosphate backbone of the nontarget DNA strand (11, Figure 6E). Our prior mutational analysis shows that highly conserved residues in the C-terminus that make hydrogen bonds to the phosphate backbone of the nontarget DNA strand are essential for strand-separation (11). Our results with R350A show that the processes of protein conformational changes and DNA strand-separation can be uncoupled. R350A is able to undergo protein conformational changes (*k*_2_) similar to WT (Figure 4A), while DNA strand separation (*k*_3_) is massively reduced in rate and amplitude (Figure 6B).

Global fitting reveals the functional consequences for R350A are primarily due to the increase in *k*_–1_ and the decrease in *K*_2_. The rate of DNA strand separation is 2.8-fold slower for R350A compared to WT (Figure 6F). However, due to the diminished PydC amplitude, the rates for this process may not be well defined. R350A generates a small amplitude of PydC signal and can form product, so we present a few possible explanations.

One possible explanation of R350A’s diminished PydC signal is that the population of GS^I^ is too low to detect PydC signal due to the destabilization of FS and GS^I^. The equilibrium constant K2 favors the reverse for R350A, while WT favors going forward in the pathway. Therefore, if GS^I^ is formed, R350A is still able to DNA strand-separate, however, the lack of PydC signal is due to the fast reverse reaction, resulting in less methylation.

Another possible explanation for R350A’s low PydC signal and slower product formation is an off-pathway non-methylatable intermediate. k_-4_ is a parameter that was included in the R350A/Cognate-DNA model due to the incomplete methylation reaction. The single-turnover methylation assay does not go to completion (Figure 6C). Therefore, *k*_–4_ was included in the model to account for the incomplete amplitude in this reaction. To reconcile this parameter, R350A could have an off-pathway intermediate that is not capable of methylation. This off-pathway intermediate would not have strand-separated DNA and may not revert to a state which can undergo methylation during the time course of the reaction, thereby explaining the diminished PydC signal and incomplete substrate turnover.

A third possible explanation for R350A’s low PydC signal and slower product formation is that this mutant undergoes an entirely different mechanism of gaining stereochemical access to the target adenine. Most *N*^6^-adenine DNA methyltransferases (unlike CcrM) solely flip the target adenine outside of the DNA helix, in a well-known process called base-flipping (43–47). R350A might be unable to separate four of the five bases of the recognition site, but may be able to base-flip the target adenine. Thus, PydC at the N-position would maintain Watson-Crick base-pairing, while the target adenine is extra-helical, explaining the diminished PydC signal, and slow rate of methylation. Interestingly, R350A is less discriminating against noncognate substrates (11), which is further evidence that strand separation is responsible for substrate discrimination.

### A new role of cofactor is proposed

S-adenosylhomocysteine (SAH) is formed as a product of the reaction and is an inhibitor of CcrM (23). SAH or the cofactor analog, sinefungin, help stabilize the strand-separated conformation (GS^II^) through interactions with Loop2B (Figure 8C). The crystal structure uses sinefungin (SFG) and our study used SAH. We show that SAH and SFG have similar effects on equilibrium Trp fluorescence changes and therefore can both be used to monitor possible changes in enzyme structure as revealed by changes in Trp fluorescence (Supplementary Figure 8). In contrast stopped-flow fluorescence in the presence of SAH shows an increase in PydC signal (Figure 8B). In the absence of SAH, we observe a small increase, followed by a smaller decrease of PydC fluorescence (Figure 8A). The decrease of the signal without SAH suggests that GS^II^ may undergo a partial reversal, or a transition to another, off pathway intermediate. Trp kinetics shows similar biphasic kinetics in the presence and absence of SAH when fit to double exponential functions (Supplementary Figure 10). This preliminary result suggests that the transition from E to GS^I^ is not entirely dependent on cofactor. Further analysis of the kinetics in the absence of cofactor may resolve the contributions of the cofactor to formation, stabilization and alignment of catalytic residues in the presence of cofactor.

The interactions seen with the cofactor in the crystal structure support our explanation of how SAH stabilizes GS^II^. Sinefungin interacts with Loop2B (Figure 8C) which consists of residues D31-E61 and the DPPY motif (D31-Y34). The DPPY motif is a conserved catalytic and SAM-interacting motif in N^6^ DNA methyltransferases that is commonly located in loop or disordered secondary structures and forms part of the active site (31). Uniquely, CcrM’s Loop2B, which contains the DPPY motif, is inserted within the strand-separated DNA (Figure 8C). We suggest that the interactions that occur at both termini of Loop2B to cofactor (SAH or sinefungin) stabilize the position of Loop2B within the strand-separated DNA, therefore stabilizing GS^II^. Other interactions from Loop2B, such as the intercalation of P45 between two thymine bases of the non-target DNA and the intraloop hydrogen bond made by Loop2B’s peptide backbone at L43 to the sidechain of K126 in Loop45 also contribute to stabilization of GS^II^ (Figure 8C, 23). These stabilizing interactions are favored only when cofactor is bound to Loop2B and are likely to provide key interactions responsible for the high specificity of CcrM.

Other cocrystal structures of MTase-dsDNA-cofactor complexes reveal similar and variable roles of the loop containing the DPPY or NPPY motif. T4Dam's DPPY is in an 8-residue loop that does not interact with DNA (40). EcoP151I’s DPPY motif is in a 19-residue loop that does not interact with DNA (56). CamA’s NPPY motif is in a 10-residue loop that makes base-specific interactions to target and non-target strand DNA bases (57). M.Taq1’s NPPY motif is in a 17-residue loop that makes base-specific interactions to three non-target strand bases (58). Thus, the cofactor-associated positioning of a loop containing the (D/N)PPY motif may not have a conserved functional role, but in the context of CcrM, the cofactor plays a unique and indirect role in stabilizing strand-separated DNA via interactions with Loop2B’s DPPY motif.

## SUMMARY

The results presented in this work contribute to our understanding of the complex mechanism that governs DNA discrimination by means of DNA strand-separation. CcrM relies on conformational intermediates in both protein and DNA, and the changes in DNA significantly contribute to substrate discrimination. We identified the conserved residues that are essential for DNA strand-separation and are found in diverse bacterial phyla and in human and animal pathogens (11). The mechanism of strand-separation and its relationship to DNA discrimination could provide insights into the similar utilization of strand-separation by enzymes that rely on recognizing dsDNA but only modify a single strand.

## Supplementary Material

gkad443_Supplemental_File

## Data Availability

No new data were generated or analysed in support of this research.
