# Correction to ‘High fidelity DNA strand-separation is the major specificity determinant in DNA methyltransferase CcrM’s catalytic mechanism’

**DOI:** 10.1093/nar/gkae195

**Published:** 2024-03-18

*Nucleic Acids Research*, Volume 51, Issue 13, 21 July 2023, Pages 6883–6898, https://doi.org/10.1093/nar/gkad443

In the original publication of this manuscript a statement of funding was incorrect. In section **Funding**, the first sentence should read: “National Science Foundation [NSF 1808775 to N.R.].” instead of: “National Science Foundation [CHE 1413722 to N.R.].”

The emendation has been made to the article.

