# Supplementary material for: High fidelity DNA strand-separation is the major specificity determinant in DNA methyltransferase CcrM’s catalytic mechanism
Source: Nucleic Acids Res. 2023 Jun 16;51(13):6883–98. doi: 10.1093/nar/gkad443 (PMC10359602; doi:10.1093/nar/gkad443)
Supplement: gkad443_Supplemental_File [file gkad443_supplemental_file.pdf]

Supplementary Information

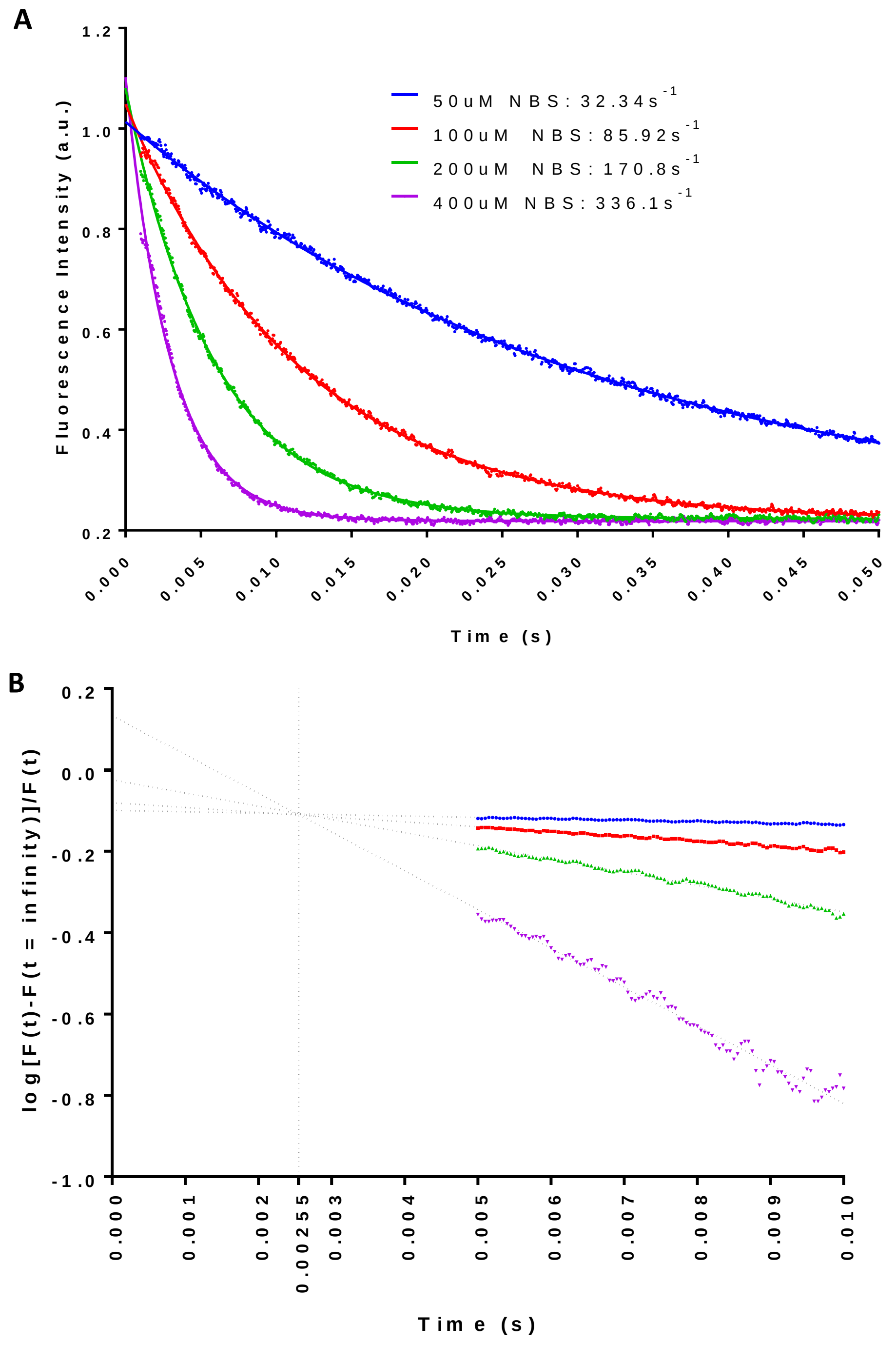

SI Figure 1. Determination of the deadtime of the stopped-flow via fluorescence methods. A. The effect of NBS concentration on the first-order rate coefficient for the quenching of N-AcTrpNH$_2$ fluorescence. Time courses of the reaction between N-AcTrpNH$_2$ (N-acetyl tryptophanamide) and NBS (N-bromosuccinimide) were collected over 50ms. Final concentrations after 1:1 mixing were: 5 μM N-AcTrpNH$_2$, 50 μM NBS (blue), 100 μM NBS (red), 200 μM NBS (green), 400 μM NBS (purple). All traces are

averages of 5 scans. Kinetics were acquired on an Applied Photophysics SX.18MV stopped-flow spectrometer (Leatherhead, UK). Excitation wavelength was 280nm with a 320nm cutoff filter. NBS concentration determines the rate of the reaction. The first 1ms of the trace was truncated to get rid of the flat regime that occurs from 0-1ms. The data were fit to a single-exponential function and the corresponding rates are listed in the figure legend. **B.** Semilogarithmic plots of the time courses of the reaction. The plot is truncated to show the data between 5-10ms to account for the linear regime of the traces. The y-axis represents a log(base10) transformation of the difference between the fluorescence intensity at time t and the fluorescence intensity at t = infinity (t = infinity is the fluorescence intensity at the equilibrium level). The traces were fit to a linear regression model and the linear fits intersect at approximately t = 2.55ms. The time between t=0 and the intersection represents the deadtime of the stopped-flow, which is approximately 2.55ms. The method used to determine the dead time was obtained from Peterman, 1979 (1).

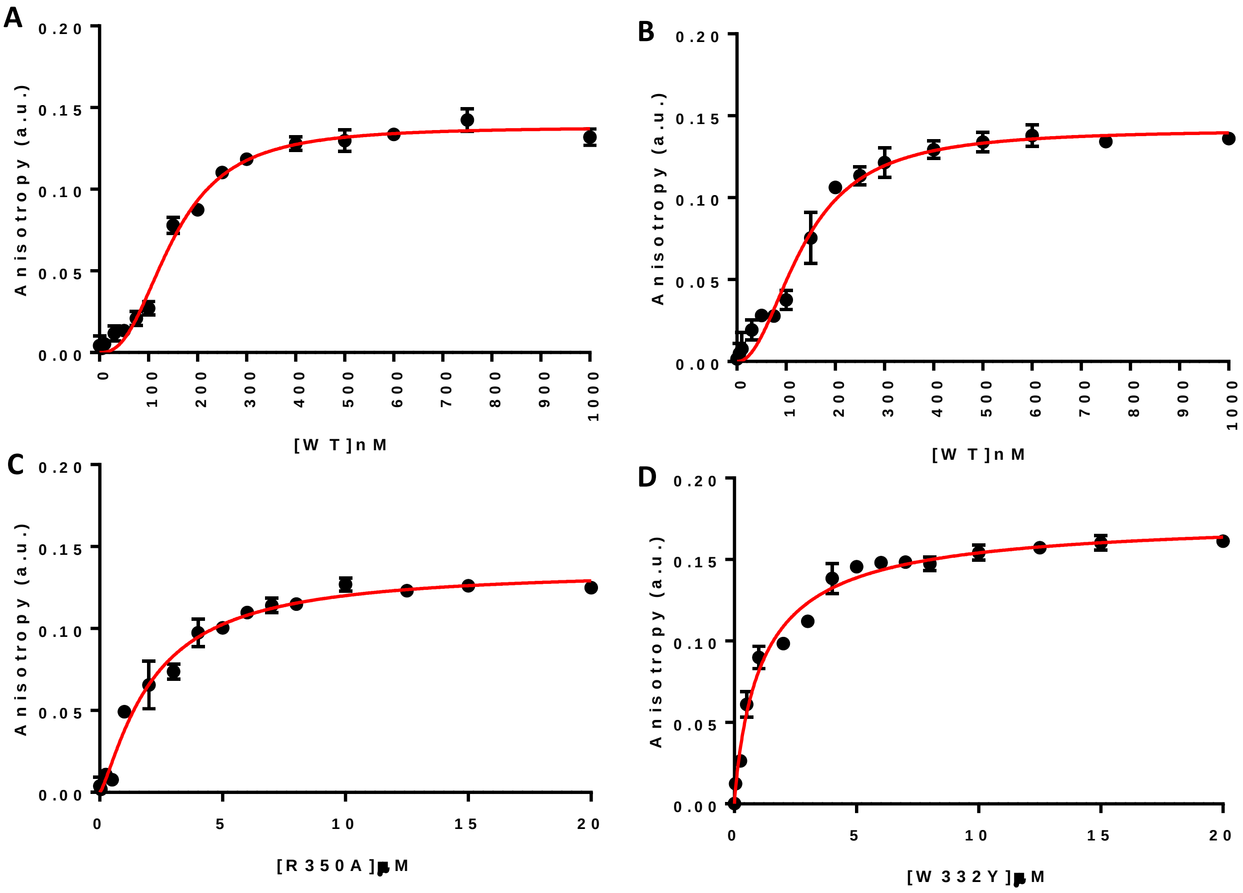

SI Figure 2. Fluorescence anisotropy to determine the K_d^{app}. Anisotropy conditions were 10nM FAM-tagged DNA, 15µM SAH, and CcrM monomer concentration (0, 10, 30, 50, 75, 100, 150, 200, 250, 300, 400, 500, 600, 750, and 1000nM). R350A and W332Y had the same conditions except for the protein concentration which ranged from 0-20µM. Data were fit in Graphpad Prism 7.00 to a specific binding with Hill slope model. $Anisotropy = Anisotropy_{max}*[CcrM]^h/(Kd^h + [CcrM]^h$. **A.** WT and cognate DNA (C1-FAM) has a K_d^{app} = 149.9 ± 5.4nM, h = 2.5. **B.** WT and noncognate DNA (NC-FAM) has a K_d^{app} = 135.2 ± 7.6nM, h = 2.1. **C.** R350A and cognate DNA (C1-FAM) has a K_d^{app} = 2111 ± 193nM, h = 1.3**. D.** W332Y and cognate DNA (C1-FAM) has a K_d^{app} = 1181 ± 135nM, h = 0.9.

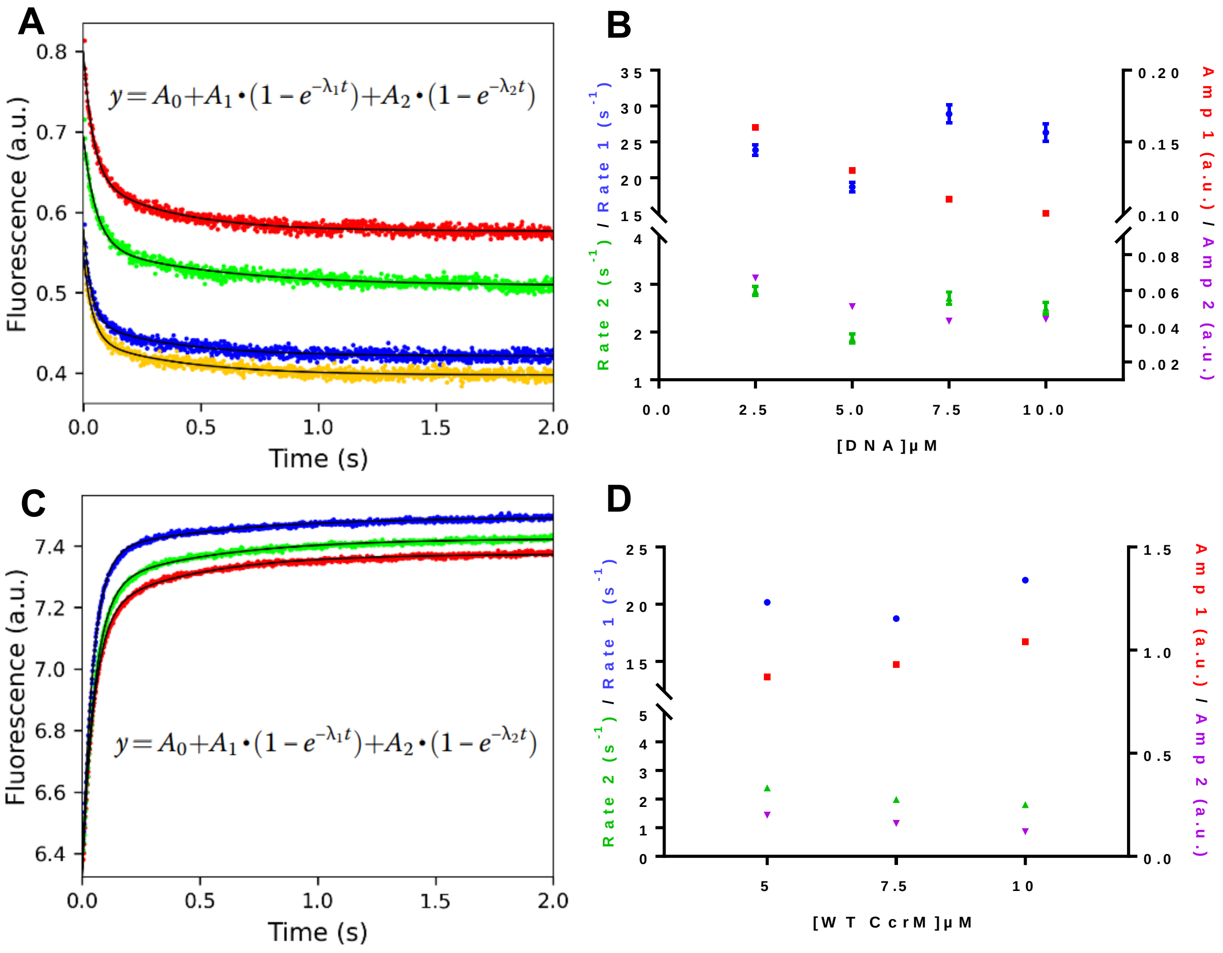

Supplemental figure 3. Conventional data fitting to double-exponential functions and the concentration dependencies on rates and amplitudes from WT / cognate DNA. A. WT/Cognate Trp data fit to a double-exponential function. **B.** Concentration-dependencies on the rates and amplitudes of the data in A. **C.** WT/Cognate DNA PdyC data fit to double-exponential functions. **D.** Concentration-dependencies on the rates and amplitudes of the data in C. The double exponential function is shown; A_0 is the initial fluorescence amplitude, A_1 is the amplitude of the first phase, λ_1 is the apparent rate of the first phase, A_2 is the amplitude of the second phase, λ_2 is the apparent rate of the second phase, and t is time in seconds. A_1, λ_1, A_2, and λ_2 correspond to Amp 1, Rate 1, Amp 2, and Rate 2, respectively.

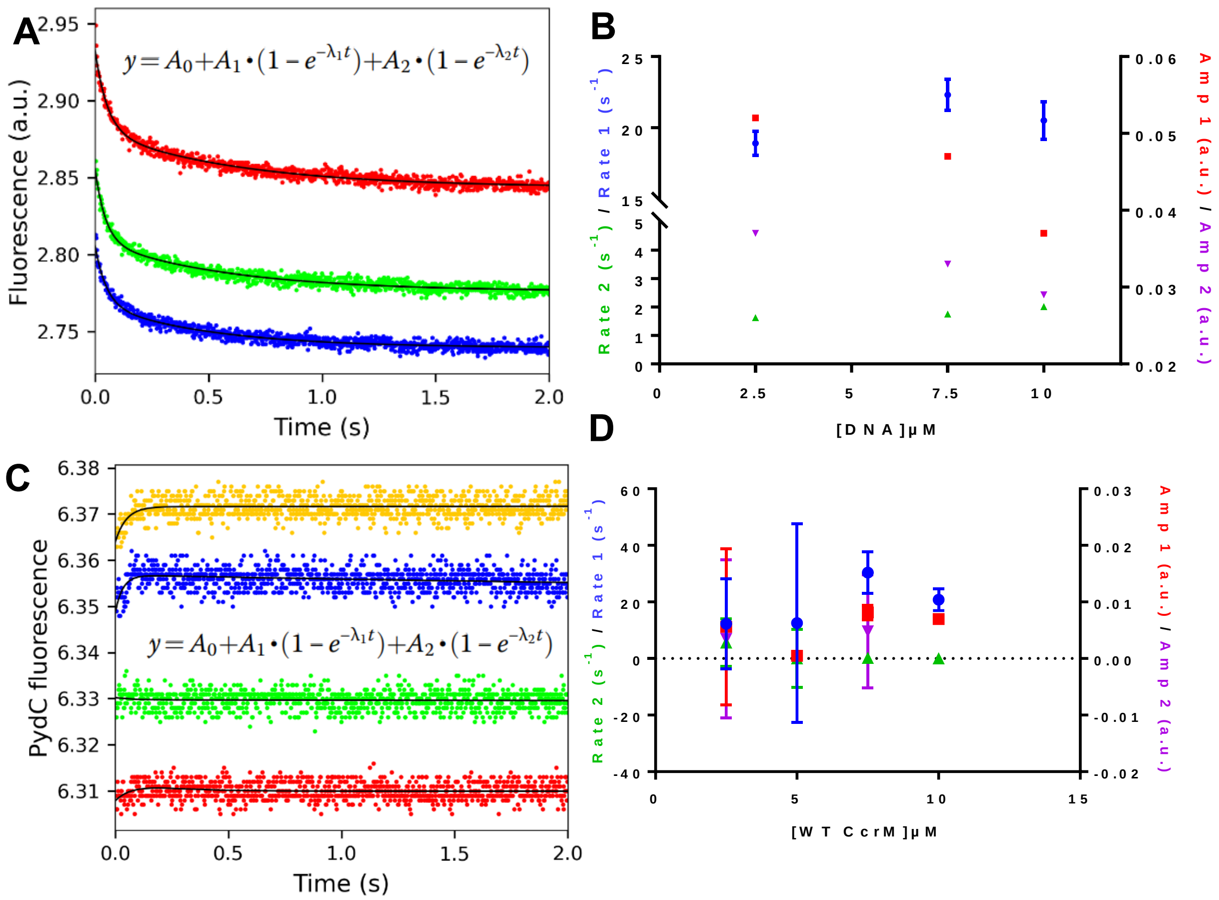

Supplemental figure 4. Conventional data fitting to double-exponential functions and the concentration dependencies on rates and amplitudes from WT / Noncognate DNA. A. WT/Noncognate Trp data fit to a double-exponential function. **B.** Concentration-dependencies on the rates and amplitudes of the data in A. **C.** WT/Noncognate DNA PdyC data fit to double-exponential functions. **D.** Concentration-dependencies on the rates and amplitudes of the data in C. The double exponential function is shown; A_0 is the initial fluorescence amplitude, A_1 is the amplitude of the first phase, λ_1 is the apparent rate of the first phase, A_2 is the amplitude of the second phase, λ_2 is the apparent rate of the second phase, and t is time in seconds. A_1, λ_1, A_2, and λ_2 correspond to Amp 1, Rate 1, Amp 2, and Rate 2, respectively.

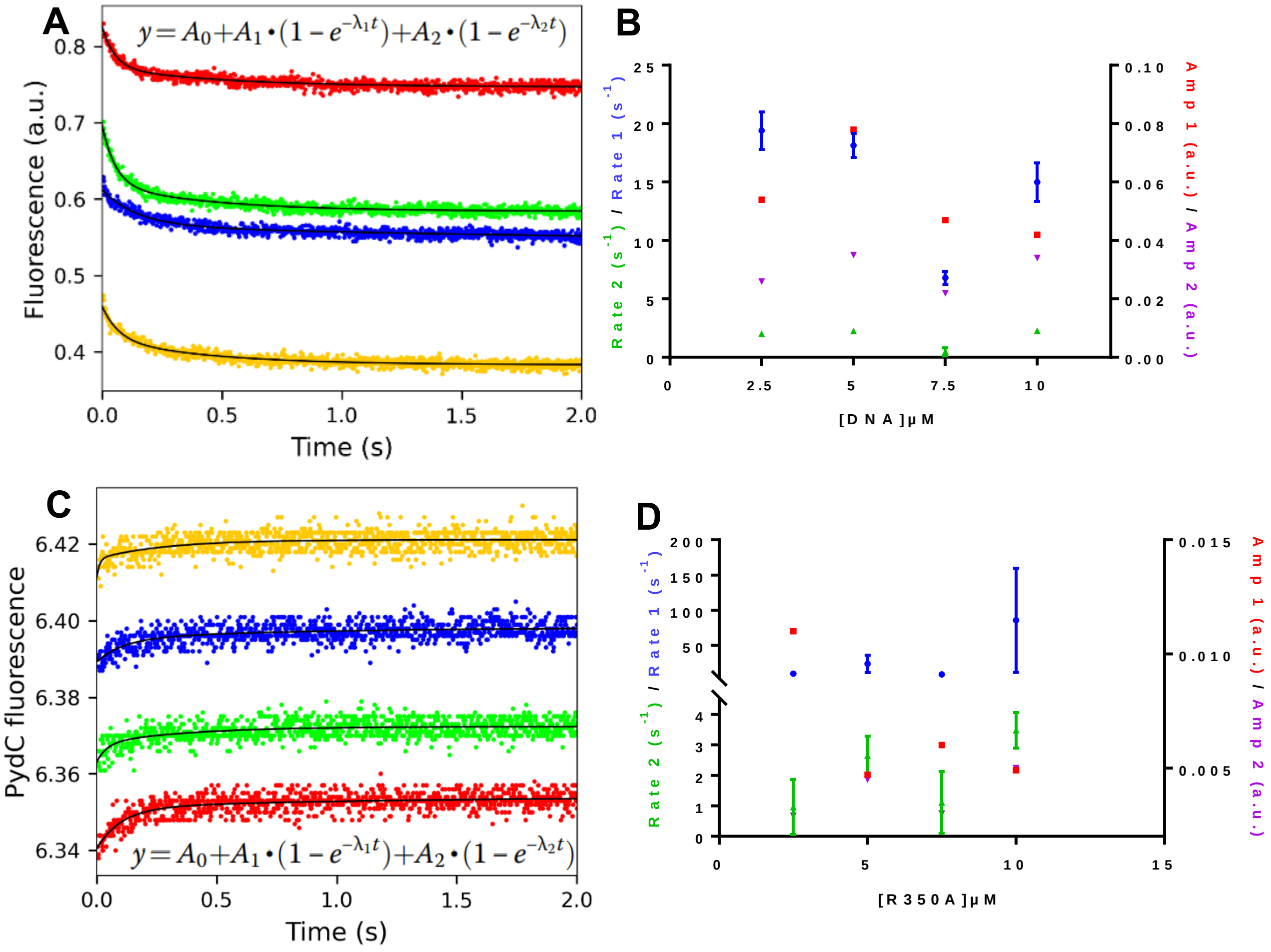

Supplemental figure 5. Conventional data fitting to double-exponential functions and the concentration dependencies on rates and amplitudes from R350A/ cognate DNA. A. R350A/Cognate Trp data fit to a double-exponential function. **B.** Concentration-dependencies on the rates and amplitudes of the data in A. **C.** R350A/Cognate DNA PdyC data fit to double-exponential functions. **D.** Concentration-dependencies on the rates and amplitudes of the data in C. The double exponential function is shown; A_0 is the initial fluorescence amplitude, A_1 is the amplitude of the first phase, λ_1 is the apparent rate of the first phase, A_2 is the amplitude of the second phase, λ_2 is the apparent rate of the second phase, and t is time in seconds. A_1, λ_1, A_2, and λ_2 correspond to Amp 1, Rate 1, Amp 2, and Rate 2, respectively.

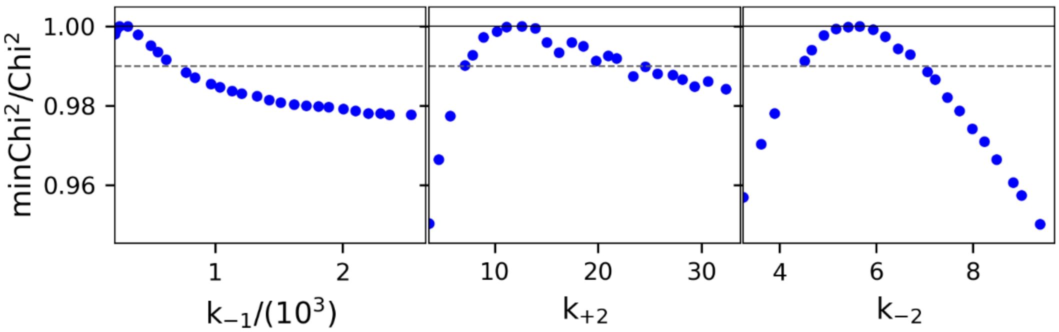

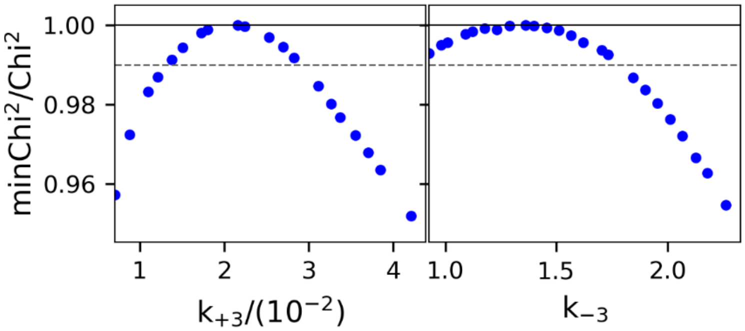

<u>**Rate**</u>	<u>**95% confidence interval**</u>
k_{-1}	185-619
k_2	7.1-22
k_{-2}	4.5-6.7
k_3	0.014-0.029
k_{-3}	0.89-1.73

Supplemental figure 6. Confidence contour analysis for the WT and Noncognate DNA model. The data represent the 1D Fitspace calculated for each rate constant. The dashed line establishes the 95% confidence interval at the 0.99 Chi^2 threshold. The 95% Chi^2 limits were calculated in Kintek Explorer and are shown in the table.

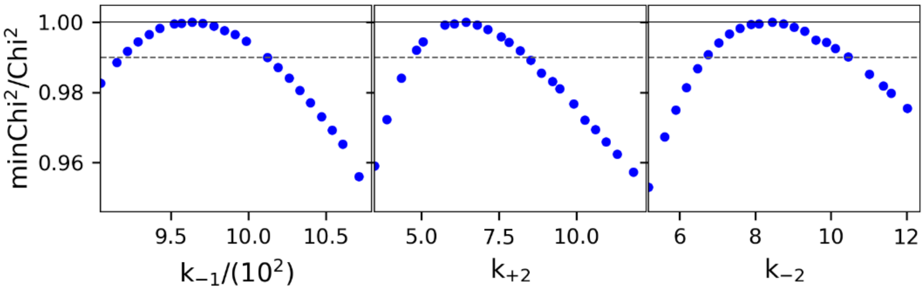

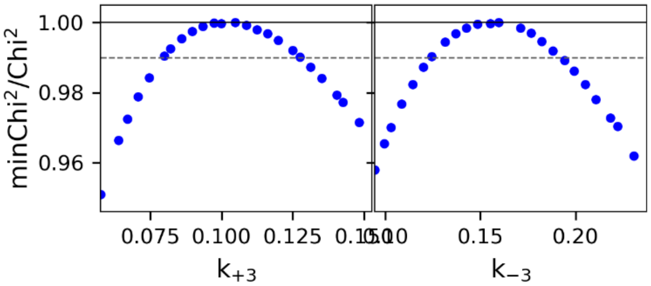

Rate	95% confidence interval
k_{-1}	922-1010
k_2	4.84-8.19
k_{-2}	6.74-10.4
k_3	0.08-0.13
k_{-3}	0.12-0.19

Supplemental figure 7. Confidence contour analysis show that the data are well-defined for the R350A and cognate DNA model. The data represent the 1D Fitspace calculated for each rate constant. The dashed line establishes the 95% confidence interval at the 0.99 Chi^2 threshold. The 95% Chi^2 limits were calculated in Kintek Explorer and are shown in the table.

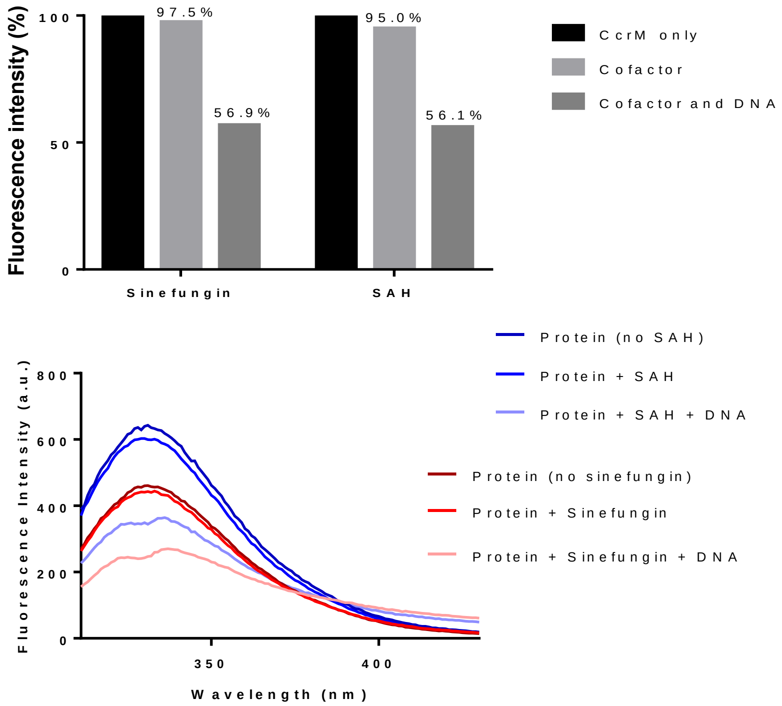

SI Figure 8. Sinefungin and SAH have a similar effect on equilibrium Trp fluorescence. CcrM Trp fluorescence is normalized to 100%. The addition of cofactor contributes to 2.5% (Sinefungin) and 5.0% (SAH). Addition of DNA and Sinefungin contributes to 43.1% of the observed effect. Addition of DNA and SAH contributes to 43.9% of the observed effect. DNA contributes to more of the observed effect than the cofactor (the latter contributes minimally to the observed change in Trp fluorescence).

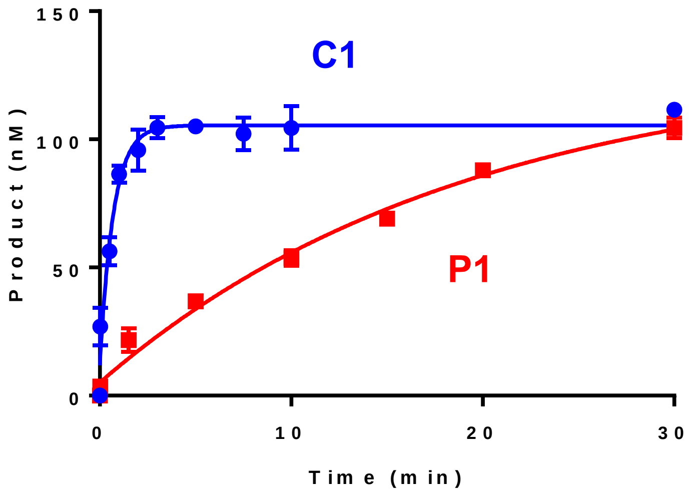

SI Figure 9. Comparison of $k_{methylation}$ for WT CcrM with cognate-DNA and PydC-cognate-DNA. Blue = Cognate DNA (C1-DNA), Red = PydC cognate DNA (P1-DNA). The conditions for both experiments consisted of WT [250nM], DNA [100nM], and SAM [15 μM]. The apparent $k_{methylation}$ with C1 DNA = 1.4 min^{-1}. The apparent $k_{methylation}$ with P1 DNA = 0.052min^{-1}.

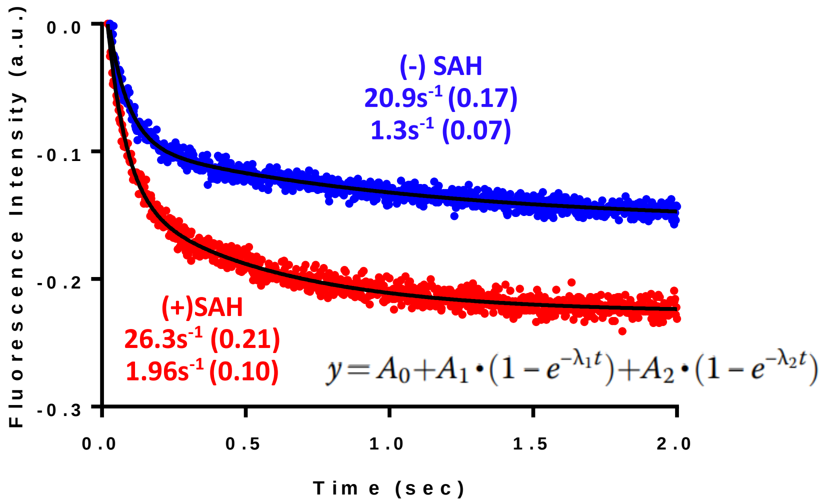

SI Figure 10. Trp kinetics are biphasic in the presence and absence of SAH. In the presence of SAH (red trace) Trp kinetics are biphasic. The apparent rate of the first phase is 26.3s^{-1} with a fluorescence amplitude of 0.21. The apparent rate of the second phase is 1.96s^{-1} with a fluorescence amplitude of 0.10. In the absence of SAH (blue trace) Trp kinetics are also biphasic. The apparent rate of the first phase is 20.9s^{-1} with a fluorescence amplitude of 0.17. The apparent rate of the second phase is 1.3s^{-1} with a fluorescence amplitude of 0.07. Conditions consisted of CcrM[500nM], C1-DNA[2.5 μM], SAH[0μM or 60μM]. The double exponential function is shown.

References

1. Peterman,B.F. (1979) Measurement of the dead time of a fluorescence stopped-flow instrument. *Analytical Biochemistry*, 93, 442-444.
